# Genome-wide survey of potato MADS-box genes reveals that StMADS1 and StMADS13 are putative downstream targets of tuberigen StSP6A

**DOI:** 10.1186/s12864-018-5113-z

**Published:** 2018-10-03

**Authors:** Huhu Gao, Ziming Wang, Silu Li, Menglu Hou, Yao Zhou, Yaqi Zhao, Guojun Li, Hua Zhao, Haoli Ma

**Affiliations:** 1 College of Agronomy, Northwest A&F University, Yangling, 712100 Shaanxi China; 2 School of Stomatology, Wuhan University, Wuhan, 430072 Hubei China

**Keywords:** Potato, MADS-box, Tuberigen, StSP6A, Tuberization

## Abstract

**Background:**

MADS-box genes encode transcription factors that are known to be involved in several aspects of plant growth and development, especially in floral organ specification. To date, the comprehensive analysis of potato MADS-box gene family is still lacking after the completion of potato genome sequencing. A genome-wide characterization, classification, and expression analysis of MADS-box transcription factor gene family was performed in this study.

**Results:**

A total of 153 MADS-box genes were identified and categorized into MIKC subfamily (MIKC^C^ and MIKC^*^) and M-type subfamily (Mα, Mβ, and Mγ) based on their phylogenetic relationships to the *Arabidopsis* and rice MADS-box genes. The potato M-type subfamily had 114 members, which is almost three times of the MIKC members (39), indicating that M-type MADS-box genes have a higher duplication rate and/or a lower loss rate during potato genome evolution. Potato MADS-box genes were present on all 12 potato chromosomes with substantial clustering that mainly contributed by the M-type members. Chromosomal localization of potato MADS-box genes revealed that MADS-box genes, mostly MIKC, were located on the duplicated segments of the potato genome whereas tandem duplications mainly contributed to the M-type gene expansion. The potato MIKC subfamily could be further classified into 11 subgroups and the TT16-like, AGL17-like, and FLC-like subgroups found in *Arabidopsis* were absent in potato. Moreover, the expressions of potato MADS-box genes in various tissues were analyzed by using RNA-seq data and verified by quantitative real-time PCR, revealing that the MIKC^C^ genes were mainly expressed in flower organs and several of them were highly expressed in stolon and tubers. StMADS1 and StMADS13 were up-regulated in the StSP6A-overexpression plants and down-regulated in the StSP6A-RNAi plant, and their expression in leaves and/or young tubers were associated with high level expression of StSP6A.

**Conclusion:**

Our study identifies the family members of potato MADS-box genes and investigate the evolution history and functional divergence of MADS-box gene family. Moreover, we analyze the MIKC^C^ expression patterns and screen for genes involved in tuberization. Finally, the StMADS1 and StMADS13 are most likely to be downstream target of StSP6A and involved in tuber development.

**Electronic supplementary material:**

The online version of this article (10.1186/s12864-018-5113-z) contains supplementary material, which is available to authorized users.

## Background

The MADS-box gene family has been extensively studied for its important roles in transcriptional regulation in eukaryotes [1–3]. The word of MADS-box is an acronym for Mini chromosome maintenance 1 (MCM1) in yeast (*Sacchromyces cerevisiae*), AGAMOUS (AG) in *Arabidopsis* (*Arabidopsis thaliana*), DEFICIENS (DEF) in snapdragon (*Antirrhinum majus*) and serum response factor (SRF) in human (*Homo sapiens*) [4–7]. MADS-box genes are characterized by N-terminal conservative MADS-box domains that are approximately 58–60 amino acids in length, which functions in combination to DNA [7, 8]. In plants, MADS-box transcription factors are involved in almost every important process during plant growth and development [9, 10].

Based on phylogenetic relationship, MADS-box gene family has been divided into two major lineages in plants, type I and type II, which were resulting from an ancestral gene duplication [11, 12]. Type I genes are also named as M-type MADS-box genes, which contain three subgroups (Mα, Mβ, and Mγ). The classical structure of M-type MADS-box genes is an N-terminal MADS domain and a relatively less conservative domain in the C-terminal [13]. In most plants, higher frequency of segmental gene duplications and weaker purifying selection result in a faster step of birth-and-death to type I genes compared to type II genes [14]. Type II MADS-box genes are also known as MIKC-type genes, which encode MEF2-like proteins [15]. In addition to the MADS domain, type II MADS-box genes contain three other domains, including intervening (I), kertain-like (K), and C-terminal (C) domains from N-terminal to C-terminal. The intervening (I) domain consists of approximately 30 amino acids and contributes to the dimerization of MADS-box proteins [16]. The kertain-like (K) domain is about 70 amino acids and more conservative than intervening (I) domain. The coiled-coil structure is significant to regulate the dimerization of MADS-box proteins. C-terminal (C) domain is a highly variable region in MADS-box proteins related to transcriptional activation and formation of protein complexes [17]. Type II MADS-box genes can be further classified into MIKC^C^ (the ‘C’ stands for ‘Classic’) and MIKC^*^ based on the variable intervening (I) domain [18]. The domain compositions of these two subfamilies in type II are quite different. MIKC^*^ subfamily exhibit a longer intervening (I) domain and less conservative kertain-like (K) domain [19]. Therefore, in early studies, MIKC^*^ subfamily was attributed into M-type MADS-box genes named Mδ [12].

The first MADS-box gene in plants was found to be related to the differentiation of flower [3]. ABCDE model had been successfully adopted to explain the determination of floral organ identity. Recent studies have also found that MIKC^C^ subfamily is related to photoperiod-regulated floral meristem identity, gametophyte development, sporophyte (diploid) generation, seed pigmentation, and embryo development [20–24]. Most of genes in ABCDE model belong to MIKC^C^ subfamily [6, 25, 26]. Besides, MADS-box genes in MIKC^C^ subfamily plays irreplaceable biological functions in the stress-responsive processes, for instance, *TaMADS2* was up-regulated in response to wheat stripe rust infection [27].

The functions of MIKC* MADS-box genes are less elucidated than those in MIKC^C^ subfamily and it is found that the heterodimers of MIKC*-type proteins are essential for the pollen maturation and pollen tube growth in *Arabidopsis* [28]. In potato, only three MADS-box genes have been previously reported, they are potato *MADS-box 1–1* (*POTM1–1*), *StMADS11*, and *StMADS16* [29, 30]. *POTM1–1* gene expression is temporally and spatially regulated in both vegetative and floral organs, transcriptional suppression of *POTM1–1* activates axillary meristem development by increasing the cytokinin levels [29, 31, 32]. *StMADS11* is expressed in all vegetative tissues of the potato plant, mainly in the stem, but not in flower organs [33]. Ectopic expression of *StMADS16* modifies the inflorescence structure by increasing both internode length and flower proliferation of the inflorescence meristems and confers vegetative features to the flower [29]. Recent study finds that FLOWERING LOCUS T in potato (StSP6A) is a mobile signal for potato tuberization. StSP6A, homologs of FT in *Arabidopsis*, is very likely to control tuberization through regulating the expressions of downstream MADS-box genes [34, 35]. Therefore, there is an urgent need to characterize the MADS-box gene family in potato and screen for MADS-box candidates involved in tuberization. The complete genome sequencing of the potato in 2011 enabled us to perform a genome-wide identification of MADS-box genes in potato [36].

In this study, multiple bioinformatics methods were applied to perform a comprehensive survey of MADS-box genes in potato. In addition, the gene structure, phylogenetic relationships, chromosomal locations, conserved motifs and tissue-specific expressions of MADS-box genes were investigated in potato. Our work would be useful in helping to establish the basic information of MADS-box genes in potato and in screening out several MADS-box genes related to tuberization and following tuber development.

## Methods

### Identification of MADS-box genes in potato

The potato genome sequence data used for the identification and annotation of *StMADS* genes was downloaded from Potato Genome Sequencing Consortium (PGSC, http://potato.plantbiology.msu.edu/). BLASTP, InerPro ID and keyword searches were performed to obtain the putative MADS-box genes in potato. First of all, the known *Arabidopsis* MADS-box protein sequences were used as query to perform BLASTP utility against the potato protein database (PGSC_DM_v3.4_pep_nonredundant.fasta) in local computer with an expected value cutoff of 1e-3. Then, InterPro ID (IPR003340) and keyword searches (MADS-box) were also applied to identify putative potato MADS-box proteins in PGSC database by online searching. All putative MADS-box sequences were collected and the redundant sequences were manually removed, the remaining candidate MADS-box sequences were submitted to NCBI Conserved Domain (CD) search (https://www.ncbi.nlm.nih.gov/Structure/cdd/wrpsb.cgi) to confirm the existence of MADS-box domain.The gene structure of MADS genes was drawn with TB tools (http://cj-chen.github.io/tbtools/) using GFF3 files downloaded from PGSC.

### Chromosomal location and gene duplication

MapChart 2.2 was exploited to draw the gene location in the physical map of potato MADS-box genes [37]. Potato MADS-box genes were named based on the position information obtained from PGSC with concerning about already reported POTM1–1 (StMADS1), StMADS11, and StMADS16. According to the nomenclature method used in rice, the remaining potato MADS-box genes were named as StMADS2 to StMADS153 followed the order of MIKC^C^, MIKC*, Mα, Mβ, and Mγ. Potato MADS-box genes without chromosomal positions were named at the last of the list. Tandem duplicated genes were determined in PGSC with a criterion that no more than five genes between two genes with high homology (> 50%). Segmental duplicated genes of potato were obtained from the Plant Genome Duplication Database (PGDD, http://chibba.pgml.uga.edu/duplication/).

### Phylogenetic and conserved motif analyses of potato MADS-box genes

Potato, rice, and *Arabidopsis* MADS-box protein sequences were aligned using ClustalX version 1.83. The phylogenetic trees were generated by using Neighbor joining (NJ) method in MEGA6.06 with bootstrap value 1000 replicates to evaluate the significance of the nodes. To ensure that the divergent domains could contribute to the topology of the NJ tree, pairwise gap deletion mode was used to construct the tree. Moreover, the potato MADS-box protein sequences were submitted to MEME (http://meme-suite.org/) to determine the conserved motif in these sequences.

### Phylogenetic analysis of MADS-box genes between potato and tomato

To investigate the phylogenetic relationships of MADS-box genes between potato and tomato, a genome-wide search against the *Solanum lycopersicum* proteome (*Solanum lycopersicum* Annotation Release 103, ftp://ftp.ncbi.nlm.nih.gov/genomes/Solanum_lycopersicum/protein/) using blastp program in dos environment of windows. The threshold of e-value was set for 1e-3. The candidate gene was submitted to InterPro to exclude genes without MADS-box domain. A phylogenetic tree was generated to determine the relationship of MADS genes in these two species using the protein sequence aligned with Muscle program in MEGA7.0 and the same method mentioned above.

### Expression analyses based on publicly available RNA-seq and microarray data

The expression patterns of potato MADS-box gene family members were determined using the data deposited in the PGSC database which derived from Illumina RNA-seq of a wide range of developmental stages [36]. The expression profiles of genes on POCI (Potato Oligo Chip Initiative) microarrays were previously performed in stolons of StSP6A-overexpression and StSP6A-RNAi plants [34]. The MADS-box genes were used as queries to search DNA probes using BLASTN and the results of MADS-box genes were used for further study with e-value set for 1e-3. The probes were selected if they were annotated as MADS-box family members by the microarray platform and were assigned to corresponding potato MADS-box genes if their identity were 100% with exceptions of single nucleotide polymorphism (SNP). The deduced FPKM value of these genes acquired from published data were normalized with Log2 to make it suitable for the further visualized in Pretty Heatmap (http://www.ehbio.com/ImageGP/index.php/Home/Index/PHeatmap.html).

### Plant materials collection and qRT-PCR

The plants of potato cultivar ‘Desire’ was cultivated in greenhouse of Northwest A&F University from March to June (23 ± 2 °C,16 h light/8 h dark). The different tissues and organs were collected at different time after sprouting. Stem, leaf, and flower were sampled at flowering, whereas stolons and young tubers were collected ten days after flowering. In addition, mature tubers were taken 90 days after sprouting. All samples were immediately frozen in liquid nitrogen and stored at -80 °C until used. Total RNA was extracted using a high purity total RNA rapid extraction kit (BioTeke, RP1202, China) and first-strand cDNA was synthesized using a ReverTra Ace Kit (TOYOBO, FSK-100, Japan) following the manufacturer’s instructions.

Primer 5.0 was used to design gene-specific primers of MADS-box in potato (Additional file 1: Table S1). Real-time quantitative RT-PCR was performed by using the SYBR green mix (KAPA, KK4601, USA) in a Real-time PCR machine (BioRad, CFX96, USA). The internal reference gene was *ef1α* and three biological replicates were used to estimate the expression level by the method of two stand curves as described previously [38].

## Results

### Identification and comparative analysis of *MADS* genes in potato

**Fig. 1:**
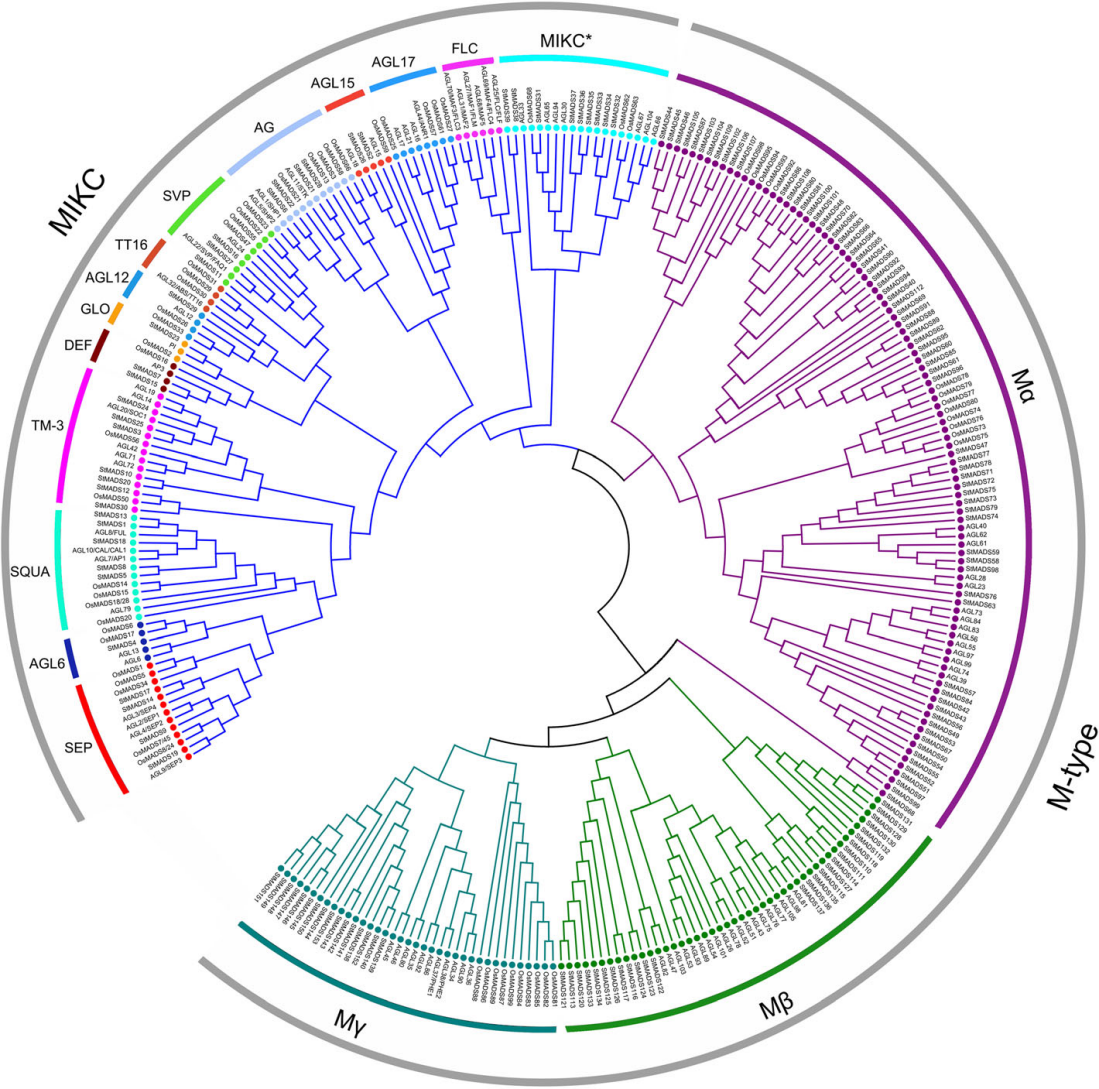
Phylogenetic tree of Arabidopsis, rice and potato MADS-box proteins. A total of 153 protein sequences of potato MADS-box genes, 89 of rice and 60 of Arabidopsis were pre-aligned by ClustalX (1.83) and used for constructing a NJ-tree in Mega 7 with 1000 replicates in bootstrap values. As is shown above, all clades are colored and arced to make it clear

Three bioinformatics methods were used to identify the MADS-box genes in potato. A local BLASTP search was performed with a cutoff e-value of 1e-3 by using the *Arabidopsis* MADS-box proteins as query, which resulted in 169 MADS-box candidates. The keyword and InterPro ID (IPR003340) searches against the PGSC website resulted in 145 and 156 MADS-box candidates, respectively. These candidates were submitted to NCBI CDD to confirm the existence of MADS-box domain. After removing the redundant sequences, 153 total MADS-box genes were found in potato. The names of *StMADS1*, *StMADS11*, and *StMADS16* were introduced by previous reports and remaining genes were named from StMADS2 to 153 (except for *StMADS11* and *StMADS16*) according to their chromosomal locations and subfamily affiliation (Table 1). Based on phylogenetic relationships of *Arabidopsis*, rice, and potato MADS-box proteins (Fig. 1), 153 potato MADS-box proteins were classified into two subfamily MIKC and M-type. In potato, the number of MADS-box genes in MIKC subfamily was 39 and this subfamily was mainly comprised of two subgroups, 30 MADS-box genes in MIKC^C^ and 9 MADS-box genes in MIKC*. The number of MADS-box genes in M-type is 114 and this subfamily contained three subgroups, 70 MADS-box genes in Mα, 28 MADS-box genes in Mβ, and 16 MADS-box genes in Mγ.

**Table 1:** The detailed information of potato MADS-box gene family. Genes with an asterisk tail means identified in previous studies

Name	Locus	Chromosomal locations	Protein (aa)	Exon	Subfamily
StMADS1^*^	PGSC0003DMG400004081	chr06 51191112–51198207	250	8	MIKC^C^
StMADS2	PGSC0003DMG401006771	chr01 64818874–64826057	287	8	MIKC^C^
StMADS3	PGSC0003DMG400000008	chr01 71388491–71392334	224	8	MIKC^C^
StMADS4	PGSC0003DMG400000136	chr01 71421222–71426490	255	8	MIKC^C^
StMADS5	PGSC0003DMG400026563	chr02 24953367–24959857	234	8	MIKC^C^
StMADS6	PGSC0003DMG400028442	chr02 31027789–31034809	248	9	MIKC^C^
StMADS7	PGSC0003DMG400003541	chr02 39864994–39869063	225	7	MIKC^C^
StMADS8	PGSC0003DMG400001378	chr02 45341112–45345702	247	8	MIKC^C^
StMADS9	PGSC0003DMG400001377	chr02 45353007–45357495	246	8	MIKC^C^
StMADS10	PGSC0003DMG401024252	chr03 41578383–41583244	119	3	MIKC^C^
StMADS11^*^	PGSC0003DMG400033570	chr01 82400439–82408299	221	9	MIKC^C^
StMADS12	PGSC0003DMG401015205	chr03 50457307–50460580	200	8	MIKC^C^
StMADS13	PGSC0003DMG400024625	chr03 54879384–54886641	248	8	MIKC^C^
StMADS14	PGSC0003DMG400024626	chr03 54896515–54901975	246	8	MIKC^C^
StMADS15	PGSC0003DMG400003709	chr04 70409561–70413264	228	7	MIKC^C^
StMADS16^*^	PGSC0003DMG400009363	chr04 64959661–64967188	235	11	MIKC^C^
StMADS17	PGSC0003DMG400028359	chr05 757295–763364	242	8	MIKC^C^
StMADS18	PGSC0003DMG400028358	chr05 766418–772401	244	8	MIKC^C^
StMADS19	PGSC0003DMG400021899	chr05 14664987–14671873	241	8	MIKC^C^
StMADS20	PGSC0003DMG400025279	chr06 36656761–36660368	111	3	MIKC^C^
StMADS21	PGSC0003DMG400005176	chr06 47282868–47291175	232	9	MIKC^C^
StMADS22	PGSC0003DMG400017295	chr07 51027282–51036001	254	6	MIKC^C^
StMADS23	PGSC0003DMG401007392	chr08 40704456–40708461	210	7	MIKC^C^
StMADS24	PGSC0003DMG400022748	chr08 53768699–53776641	224	7	MIKC^C^
StMADS25	PGSC0003DMG400010263	chr10 38698247–38706939	214	6	MIKC^C^
StMADS26	PGSC0003DMG400023729	chr10 57811655–57814557	229	7	MIKC^C^
StMADS27	PGSC0003DMG400016203	chr11 2317625–2323465	238	8	MIKC^C^
StMADS28	PGSC0003DMG400025525	chr11 16127866–16137240	232	7	MIKC^C^
StMADS29	PGSC0003DMG401018787	chr11 16991875–16995488	116	4	MIKC^C^
StMADS30	PGSC0003DMG400001938	chr00 12593321–12596548	188	2	MIKC^C^
StMADS31	PGSC0003DMG400019525	chr03 55865548–55867778	142	3	MIKC^*^
StMADS32	PGSC0003DMG400038617	chr04 31598124–31598495	84	2	MIKC^*^
StMADS33	PGSC0003DMG400036414	chr04 52111872–52112228	82	2	MIKC^*^
StMADS34	PGSC0003DMG400044568	chr04 52327361–52331720	158	3	MIKC^*^
StMADS35	PGSC0003DMG400045842	chr06 35336837–35338244	151	5	MIKC^*^
StMADS36	PGSC0003DMG401026197	chr07 47364697–47367540	155	6	MIKC^*^
StMADS37	PGSC0003DMG403026197	chr07 47437818–47445958	94	9	MIKC^*^
StMADS38	PGSC0003DMG400017759	chr12 55492383–55493047	108	2	MIKC^*^
StMADS39	PGSC0003DMG400017760	chr12 55554242–55562194	225	3	MIKC^*^
StMADS40	PGSC0003DMG400038539	chr01 14769002–14769334	110	1	Mα
StMADS41	PGSC0003DMG400045528	chr01 40043124–40043513	129	1	Mα
StMADS42	PGSC0003DMG400011328	chr01 57376759–57377670	303	1	Mα
StMADS43	PGSC0003DMG400011316	chr01 57795443–57796789	421	1	Mα
StMADS44	PGSC0003DMG400006290	chr01 60247610–60248203	197	1	Mα
StMADS45	PGSC0003DMG400041956	chr01 60265275–60265868	197	1	Mα
StMADS46	PGSC0003DMG400046706	chr01 60293060–60293646	189	2	Mα
StMADS47	PGSC0003DMG400000062	chr01 72596658–72597790	143	1	Mα
StMADS48	PGSC0003DMG400040999	chr01 75519222–75519740	172	1	Mα
StMADS49	PGSC0003DMG400022465	chr01 75665917–75666411	495	1	Mα
StMADS50	PGSC0003DMG400022464	chr01 75669936–75670403	155	1	Mα
StMADS51	PGSC0003DMG400037739	chr01 75677724–75678191	155	1	Mα
StMADS52	PGSC0003DMG400022462	chr01 75682108–75682572	154	1	Mα
StMADS53	PGSC0003DMG400022460	chr01 75693397–75693888	163	1	Mα
StMADS54	PGSC0003DMG400044662	chr01 75699473–75699940	155	1	Mα
StMADS55	PGSC0003DMG400042000	chr01 75715667–75716134	155	1	Mα
StMADS56	PGSC0003DMG400035140	chr01 75719828–75720178	116	1	Mα
StMADS57	PGSC0003DMG400005119	chr01 83295937–83296952	213	1	Mα
StMADS58	PGSC0003DMG400044387	chr01 83297295–83297960	221	1	Mα
StMADS59	PGSC0003DMG400005131	chr01 83300154–83300747	197	1	Mα
StMADS60	PGSC0003DMG400046652	chr02 14861994–14862299	101	1	Mα
StMADS61	PGSC0003DMG400038730	chr02 15687958–15688452	164	1	Mα
StMADS62	PGSC0003DMG400013012	chr02 22892178–22892726	182	1	Mα
StMADS63	PGSC0003DMG400039595	chr02 25493501–25494016	171	1	Mα
StMADS64	PGSC0003DMG400042442	chr03 3618726–3619148	140	1	Mα
StMADS65	PGSC0003DMG400037384	chr03 3660635–3661183	182	1	Mα
StMADS66	PGSC0003DMG400045551	chr03 4972791–4973312	173	1	Mα
StMADS67	PGSC0003DMG400046651	chr03 13264973–13265449	158	1	Mα
StMADS68	PGSC0003DMG400037338	chr04 750699–751226	175	1	Mα
StMADS69	PGSC0003DMG400019742	chr04 9343623–9346031	90	6	Mα
StMADS70	PGSC0003DMG400040226	chr04 21299801–21300325	174	1	Mα
StMADS71	PGSC0003DMG400041035	chr04 27499096–27499626	176	1	Mα
StMADS72	PGSC0003DMG400036160	chr04 27559777–27560277	166	1	Mα
StMADS73	PGSC0003DMG400044513	chr04 27753241–27753636	131	1	Mα
StMADS74	PGSC0003DMG400040625	chr04 27761388–27761732	114	1	Mα
StMADS75	PGSC0003DMG400035221	chr04 27826362–27826868	168	1	Mα
StMADS76	PGSC0003DMG400036006	chr04 28232462–28232779	105	1	Mα
StMADS77	PGSC0003DMG400034832	chr04 28463661–28464235	164	2	Mα
StMADS78	PGSC0003DMG400040127	chr04 28566215–28566718	167	1	Mα
StMADS79	PGSC0003DMG400045239	chr04 29232278–29232646	122	1	Mα
StMADS80	PGSC0003DMG400042688	chr04 49390684–49391226	180	1	Mα
StMADS81	PGSC0003DMG400044761	chr04 49734932–49735474	180	1	Mα
StMADS82	PGSC0003DMG400039153	chr04 54460837–54461322	161	1	Mα
StMADS83	PGSC0003DMG400037581	chr04 54575543–54576028	161	1	Mα
StMADS84	PGSC0003DMG400024799	chr04 58551552–58552247	231	1	Mα
StMADS85	PGSC0003DMG400040743	chr06 10343031–10343519	162	1	Mα
StMADS86	PGSC0003DMG400040848	chr06 45773429–45773971	180	1	Mα
StMADS87	PGSC0003DMG400043595	chr07 15176598–15177149	183	1	Mα
StMADS88	PGSC0003DMG400004482	chr08 38320573–38321088	171	1	Mα
StMADS89	PGSC0003DMG400029147	chr08 38625243–38625695	150	1	Mα
StMADS90	PGSC0003DMG400039537	chr09 4843300–4843812	170	1	Mα
StMADS91	PGSC0003DMG400036125	chr09 6381512–6381946	144	1	Mα
StMADS92	PGSC0003DMG400043218	chr09 8426313–8426747	144	1	Mα
StMADS93	PGSC0003DMG400038182	chr09 8460217–8460714	165	1	Mα
StMADS94	PGSC0003DMG400043614	chr09 28872055–28872590	149	2	Mα
StMADS95	PGSC0003DMG400040285	chr09 45285005–45285310	101	1	Mα
StMADS96	PGSC0003DMG400037372	chr09 45311443–45311877	144	1	Mα
StMADS97	PGSC0003DMG400019393	chr10 6655038–6655631	173	1	Mα
StMADS98	PGSC0003DMG400043633	chr10 13335280–13335894	204	1	Mα
StMADS99	PGSC0003DMG400035501	chr10 16298995–16299315	106	1	Mα
StMADS100	PGSC0003DMG400042890	chr10 43539248–43539784	178	1	Mα
StMADS101	PGSC0003DMG400039674	chr10 43571982–43572518	178	1	Mα
StMADS102	PGSC0003DMG400036190	chr11 36057399–36057959	186	1	Mα
StMADS103	PGSC0003DMG400047285	chr11 36128116–36128568	150	1	Mα
StMADS104	PGSC0003DMG400039835	chr11 36737512–36737970	152	1	Mα
StMADS105	PGSC0003DMG400043978	chr11 41767865–41768500	211	1	Mα
StMADS106	PGSC0003DMG400038207	chr11 41778980–41779333	117	1	Mα
StMADS107	PGSC0003DMG400001143	chr11 41867026–41867556	176	1	Mα
StMADS108	PGSC0003DMG400044457	chr12 52437112–52437654	180	1	Mα
StMADS109	PGSC0003DMG400046733	chr00 38424953–38425450	165	1	Mα
StMADS110	PGSC0003DMG400046736	chr01 44165290–44165835	181	1	Mβ
StMADS111	PGSC0003DMG400037342	chr01 60364849–60365394	181	1	Mβ
StMADS112	PGSC0003DMG400024916	chr01 80241931–80242893	320	1	Mβ
StMADS113	PGSC0003DMG400024902	chr01 80529617–80530232	139	1	Mβ
StMADS114	PGSC0003DMG400005968	chr04 1911128–1911634	168	1	Mβ
StMADS115	PGSC0003DMG400035463	chr05 9324497–9325042	181	1	Mβ
StMADS116	PGSC0003DMG400007682	chr05 9990538–9991511	157	1	Mβ
StMADS117	PGSC0003DMG400018623	chr05 10026168–10026958	219	1	Mβ
StMADS118	PGSC0003DMG400041411	chr05 51418059–51418661	200	1	Mβ
StMADS119	PGSC0003DMG400047062	chr06 8761350–8762057	235	1	Mβ
StMADS120	PGSC0003DMG400026582	chr06 44289139–44289651	131	1	Mβ
StMADS121	PGSC0003DMG400033092	chr06 52148889–52149384	131	1	Mβ
StMADS122	PGSC0003DMG400027886	chr07 39107047–39108380	299	2	Mβ
StMADS123	PGSC0003DMG400027613	chr11 12890859–12892025	388	1	Mβ
StMADS124	PGSC0003DMG400035247	chr11 13686758–13687444	228	1	Mβ
StMADS125	PGSC0003DMG400026881	chr11 17707080–17707472	130	1	Mβ
StMADS126	PGSC0003DMG400036234	chr11 29461097–29461483	128	1	Mβ
StMADS127	PGSC0003DMG400044576	chr11 40025123–40025641	172	1	Mβ
StMADS128	PGSC0003DMG400044231	chr12 10345869–10346366	165	1	Mβ
StMADS129	PGSC0003DMG400035476	chr12 10440142–10440801	219	1	Mβ
StMADS130	PGSC0003DMG400034608	chr12 10457590–10458198	202	1	Mβ
StMADS131	PGSC0003DMG400045649	chr12 10473059–10473718	219	1	Mβ
StMADS132	PGSC0003DMG400044285	chr12 11631459–11632118	219	1	Mβ
StMADS133	PGSC0003DMG400016835	chr00 15615908–15616303	131	1	Mβ
StMADS134	PGSC0003DMG400016838	chr00 15735051–15735446	131	1	Mβ
StMADS135	PGSC0003DMG400005533	chr00 27564359–27564892	177	1	Mβ
StMADS136	PGSC0003DMG400039981	chr00 27565967–27566461	164	1	Mβ
StMADS137	PGSC0003DMG400005534	chr00 27590544–27591083	179	1	Mβ
StMADS138	PGSC0003DMG400036527	chr01 45487714–45488157	147	1	Mγ
StMADS139	PGSC0003DMG400024023	chr01 45617221–45618372	383	1	Mγ
StMADS140	PGSC0003DMG400024024	chr01 45621657–45622640	327	1	Mγ
StMADS141	PGSC0003DMG400039025	chr03 5616516–5617253	245	1	Mγ
StMADS142	PGSC0003DMG400030053	chr04 20856443–20857615	390	1	Mγ
StMADS143	PGSC0003DMG400034827	chr04 23826227–23827363	378	1	Mγ
StMADS144	PGSC0003DMG400038060	chr05 38909497–38910506	213	2	Mγ
StMADS145	PGSC0003DMG400040524	chr05 39047174–39048184	336	1	Mγ
StMADS146	PGSC0003DMG400045326	chr05 39101622–39102656	344	1	Mγ
StMADS147	PGSC0003DMG400044997	chr05 39139534–39142691	349	2	Mγ
StMADS148	PGSC0003DMG400044633	chr05 41389552–41390400	282	1	Mγ
StMADS149	PGSC0003DMG400039358	chr05 43411054–43411578	174	1	Mγ
StMADS150	PGSC0003DMG400041221	chr05 43419172–43421058	259	2	Mγ
StMADS151	PGSC0003DMG400038225	chr05 43446588–43447571	327	1	Mγ
StMADS152	PGSC0003DMG400040157	chr07 41201874–41202581	235	1	Mγ
StMADS153	PGSC0003DMG400028638	chr12 50083239–50084024	261	1	Mγ

MIKC subfamily members are about 200 amino acid in length and contain more exons than those of M-type subfamily (Table 1). The MIKC family members have an average of 6.4 exon number, and 86.7% of them contain more than 5. But for the M-type, most of them (106 of 114) have only one exon (Table 1). These results about exon number in MADS-box genes are similar to those have been reported in *Arabidopsis*, rice, cucumber, and apple. The exon-intron structures of MIKC members are more complex than those of M-type.

The total number of MADS-box genes in 10 species that had been previously reported was quite different (Table 2) [12, 39–49]. The reported numbers of MADS-box genes were from 43 to 167, which were positively correlated to corresponding genome size except for that of *Arabidopsis* (Table 2). Generally, MIKC subfamily consisted of more members than M-Type subfamily as reported in previous studies, but we found that the number of M-type MADS-box genes (114) is approximately three times to that of MIKC MADS-box genes (39) in potato (Table 2). In the other nine species, the number of MIKC subfamily members was close to or more than that of M-type subfamily members.

**Table 2:** The number of MADS genes in thirteen plant species

Species	Genome size (Mb)	Total MADS genes	Total MIKC	MIKC^C^	MIKC^*^	Total M-type	Mα	Mβ	Mγ
*Cucumis sativus*	350	43	33	30	3	10	5	2	3
*Brachypodium distachyon*	260	57	39	32	7	18	9	7	2
*Sesamum indicum*	340	57	33	28	5	24	14	0	10
*Oryza sativa*	466	75	44	38	6	32	13	9	10
*Prunus mume*	280	80	37	32	5	43	20	14	9
*Vitis vinifera*	490	90	48	42	6	42	23	0	19
*Populus trichocarpa*	480	105	64	55	9	41	23	12	6
*Arabidopsis thaliana*	125	106	45	39	6	61	25	20	16
*Solanum lycopersicum*	900	107	53	41	12	54	34	6	14
*Raphanus raphanistrum*	253	144	76	70	6	68	31	12	25
*Malus domestica*	1874	146	92	75	17	54	22	8	24
*Solanum tuberosum*	844	153	39	30	9	114	70	28	16
*Brassica rapa*	485	167	100	89	11	67	29	16	22

### Chromosomal distribution and duplication events of *StMADS* genes

**Fig. 2:**
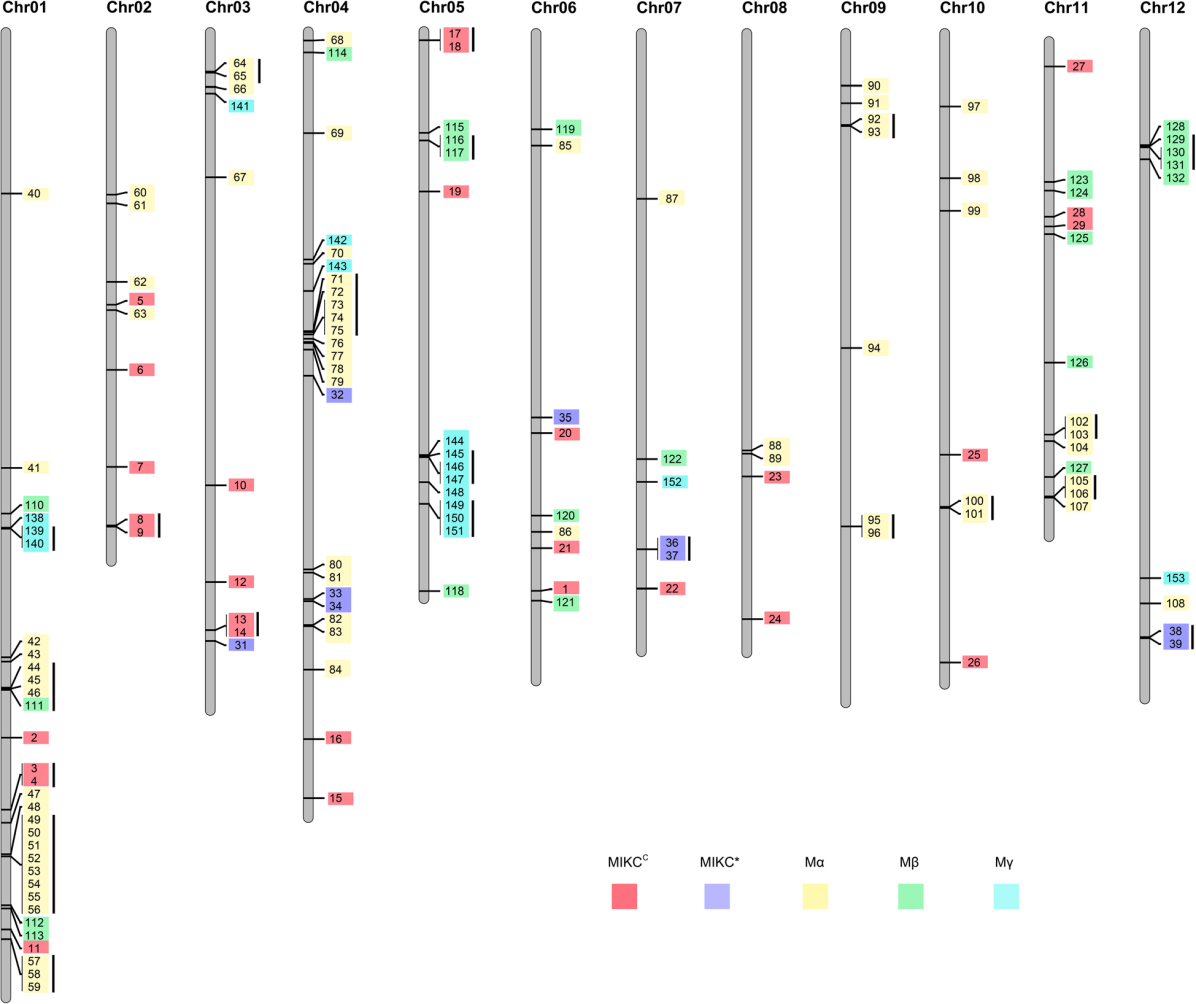
Physical map of 153 MADS-box genes at 12 potato chromosomes. Different subfamilies are shown in different colors. Genes covered with a single line means a tandem duplication gene group

The MapChart software was used to map the physical position of MADS-box genes on 12 chromosomes of potato, which would be helpful for us to perform further study of function of MADS-box genes in potato (Fig. 2). Based on the information of chromosomal locations of potato MADS-box genes, it was found that seven genes were not localized to the chromosomes of potato, five of which belonged to the M-type subfamily (Table 1). The rest MADS-box genes (146) were distributed on the 12 chromosomes and the top five chromosomes with more MADS-box genes are Chr01 (31 genes), Chr04 (25 genes), Chr05 (15 genes), Chr11 (14 genes), and Chr03 (10) (Fig. 2).

**Fig. 3:**
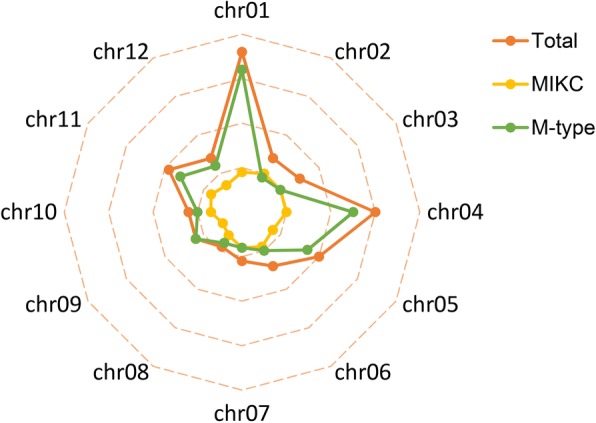
Number of MAD-box genes at 12 potato chromosomes. Different color represents different group

To further explore the distribution patterns of MADS-box genes, a radar map was exhibited to show the distributions of each subfamily in 12 chromosomes. It was found that substantial clustering was detected in each of at least four chromosomes which was mainly contributed by the gene number of M-type subfamily rather than MIKC subfamily, implying there may be a selective expansion pattern mainly happened in the M-type subfamily (Fig. 3). The MADS-box genes belong to MIKC subfamily distributed on all chromosomes except on Chr09 (Fig. 2). For the M-type MADS-box genes, 52.9% MADS-box genes of the Mα subgroup was clustered on Chr01 and Chr04.

**Fig. 4:**
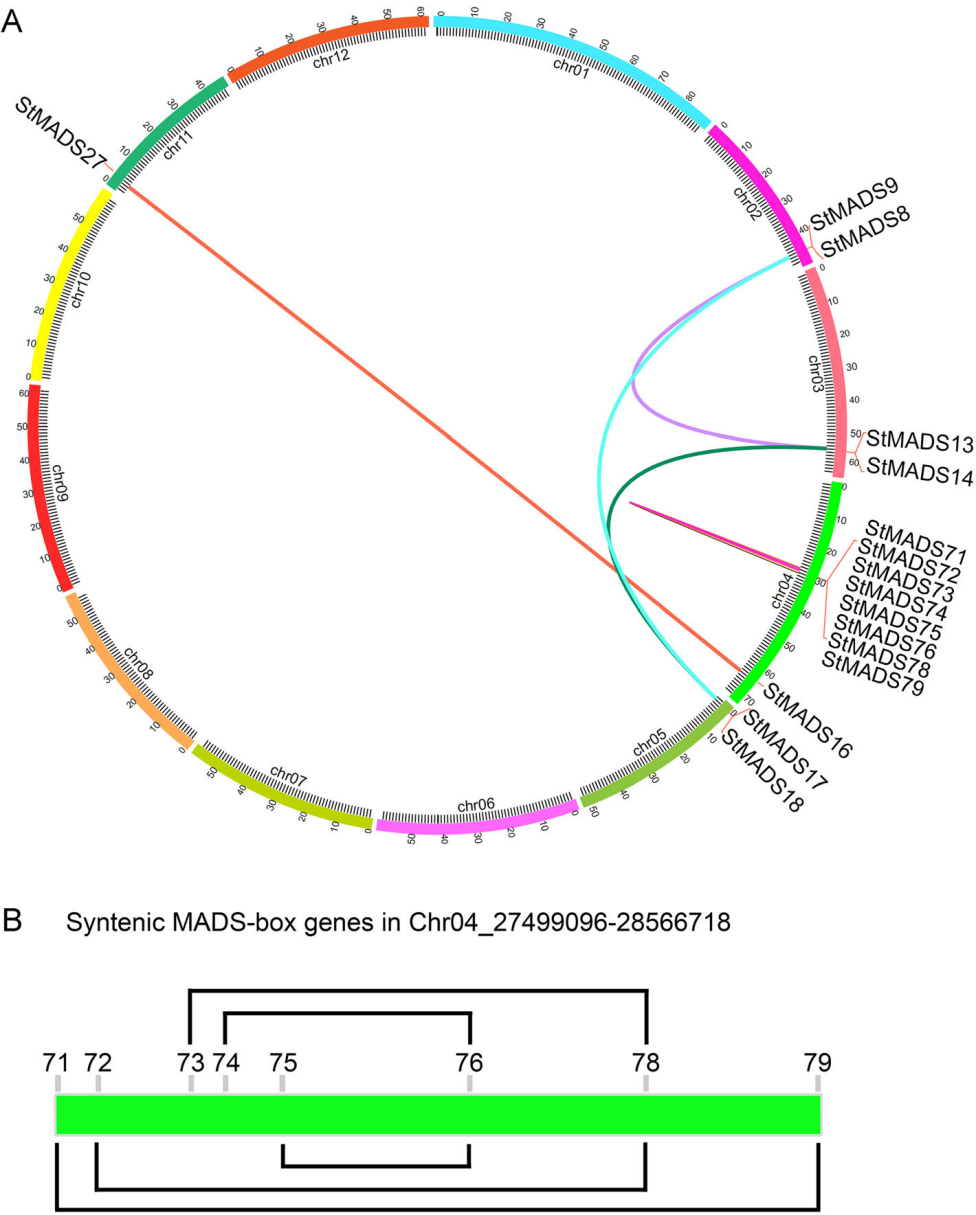
Segmental duplication genes in 12 potato chromosomes. Genes linked with a line shows a pair of segmental duplicated genes. **a** Segmental gene pairs between 12 chromosomes. **b** Micro-syntenic map in Chr04_27,499,096–28,566,718

Moreover, the gene duplication events in the MADS-box gene family were analyzed and it was found that 47.7% (73 of 153) MADS-box genes derived from gene duplications (Figs. 2 and 4a). Tandem duplicated genes were mainly located on chromosome 1 and chromosome 5, accounting for about 51.9% of tandem duplicated genes. 78.9% (45 of 57) tandem duplicated genes of belonged to the M-type, indicating that tandem duplications played an important role in the expansion of M-type family genes. 31.5% (12 of 38) genes of the MIKC subfamily were resulted from tandem duplications. Interestingly, it was found that tandem duplications could occur between different subgroups (e.g. StMADS44–46 belonged to Mα and StMADS111 belonged to Mβ), indicating that gene duplication not only contributed to the expansion of MADS-box gene family but also lead to functional diversifications.

Compared with tandem duplications, segmental duplications only accounted for 10.5% of the total MADS-box genes in potato (Fig. 4a). 26.7% (8 of 30) of MIKC^C^ MADS-box genes were resulted from segmental duplications. Those genes were located in Chr02 (two genes), Chr03 (two genes), Chr04 (one genes), Chr05 (two genes), and Chr11 (one genes). We found, interestingly, three copies of the segmental duplicated gene pair (*StMADS17* and *18*, *StMADS13* and *14*, *StMADS8* and *9*), among which *StMADS9*, *14* and *17* are from SEP group, while *StMADS8*, *13* and *18* from SQUA group. Moreover, the Mα subfamily members *StMADS71*–*79* located in chr04 were segmental duplicated genes, which shows a cluster in the physical map (Fig. 4b). It clearly shows that there probably a chromosome doubling event in chr04 in the process of potato evolution, which contribute greatly to the expansion of Mα type MADS-box genes.

### Phylogenetic relationships and conversed motifs of StMADS proteins

An unrooted tree was built based on the full-length amino acid sequences of 153 potato, 89 *Arabidopsis*, and 60 rice MADS-box proteins using MEGA6.0 software (Fig. 1). StMADS proteins can be classified into two major subfamilies, MIKC (also known as type II, 39 genes) and M-type (also known as type I, 114 genes), based on the phylogenetic tree. MIKC subfamily can be further divided into MIKC^C^ (30 genes) and MIKC* (9 genes), whereas M-type contains Mα (70), Mβ (28) and Mγ (16). According to the classification method defined in *Malus domestica*, *Oryza sativa*, and *Brassica rapa*, MIKC^C^ subgroup was organized into 13 clades. Interestingly, potato MADS-box genes were absent in the FLC-like, AGL15-like, and TT16-like clades. Subsequently, potato MIKC^C^ subgroup consisted of ten clades. TM3-like clade was the largest clade containing seven StMADS proteins. The orthologous and paralogous relationships of MADS-box proteins are analyzed in potato, rice, and *Arabidopsis*, it was found that most of the M-type subfamily members were concentrated in a cluster, namely all of these homologous MADS-box proteins are paralogous genes. These results indicated that the MADS-box gene family was formed in an ancestral species before the divergence of monocotyledonous and dicotyledonous plants, which was consistent with the results of previous studies [12, 39–52]. Moreover, orthologous pairs come from the MIKC family were with relatively high homology, indicating that the functions of MIKC family genes were relatively conservative in the evolutionary process.

**Fig. 5:**
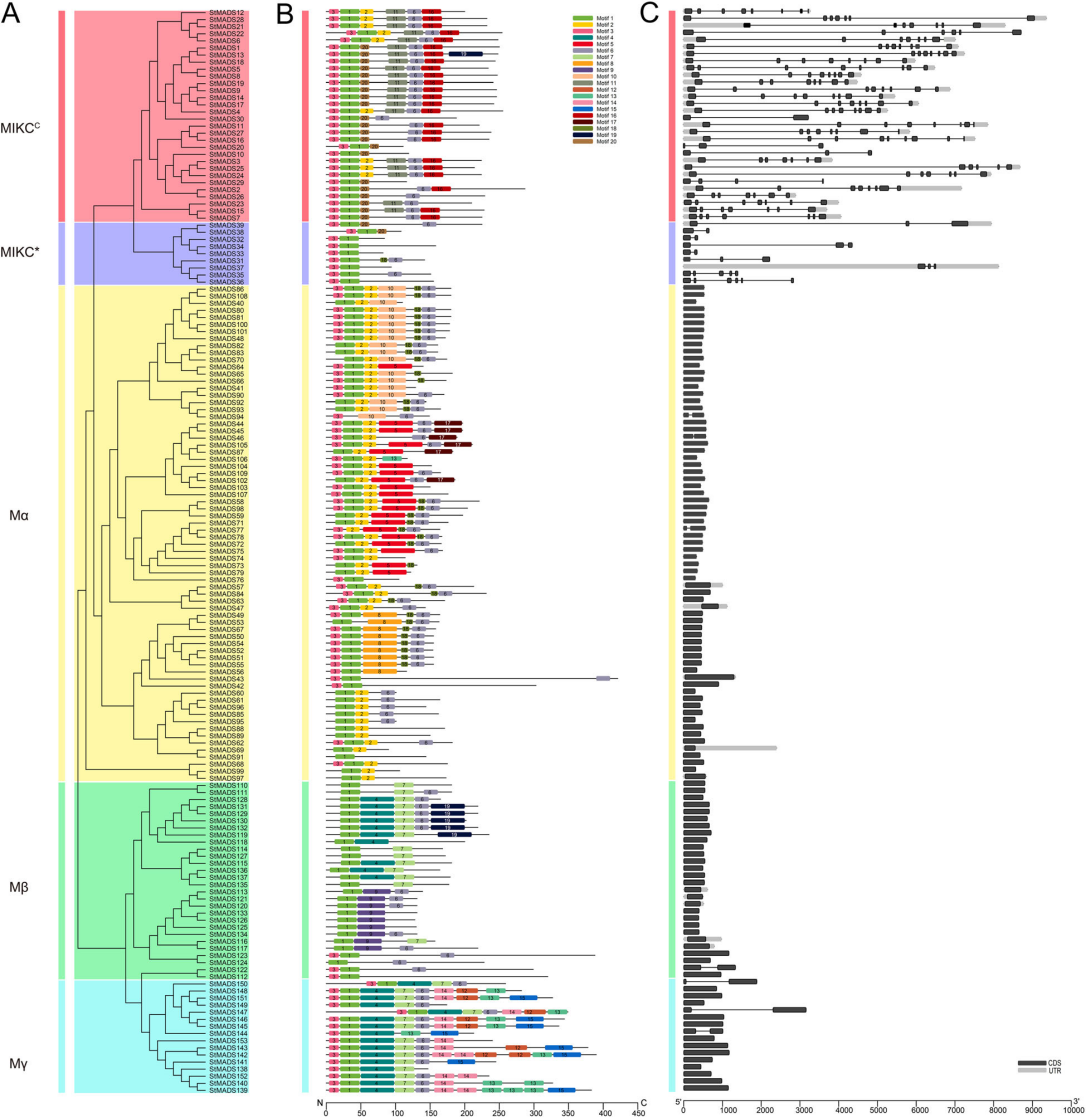
Phylogenetic relationships, conserved motif and gene structure of potato MADS genes. **a** The NJ-tree of 153 potato MADS-box genes, constructed with the same method mentioned above. **b** Conserved motif analysis of 153 MADS-box genes. Rounded rectangle with different colors represent different motif. **c** Gene structure of MADS-box genes. Exon and UTR are box colored with black and grey respectively, among which the black line represent introns

Similarly, an unrooted tree was also built based on the full-length amino acid sequences of 153 potato MADS-box proteins, which could be partitioned into MIKC^C^, MIKC*, Mα, Mβ, and Mγ with good supporting values (Fig. 5a). To further analyze the motif compositions of potato MADS-box proteins, MEME online software was used to analyze the conserved motifs (Fig. 5b). The number of conserved motif was set to 20, where motifs 1, 2, 3, and 20 were located at MADS-box domains and motifs 6, 11 and 16 were located at K domain. Moreover, motifs 4, 5, and 7–10 represented coil regions and low complexity regions. Besides, the rest motifs were less conservative and only appeared in several MADS-box proteins. As shown in Fig. 4, MIKC^C^ subgroup contains seven conservative motifs 1, 2, 3, 6, 11, 16, and 20 and motifs 11 and 16 belonging to K domain only existed in this subgroup. The MIKC* subgroup contains fewer motif varieties, mainly motifs 1 and 3, and some of them had motifs 6 and 20 similar to MIKC. Specifically, StMADS31 had motif 18 adjacent to motif6 which was similar to the members belonging to subgroup Mα. These results showed that the MADS-box proteins included in the same clade in the phylogenetic tree have almost identical motif distribution types. Moreover, the structure of MIKC* had both characteristics of M-type and MIKC^C^, but in *Arabidopsis* the subgroup MIKC* was attributed to Mδ, which were attributed to MIKC group later in the analyses of MADS-box families of other species [12, 39–52].

### Phylogenetic relationship of MADS-box genes of potato and tomato

**Fig. 6:**
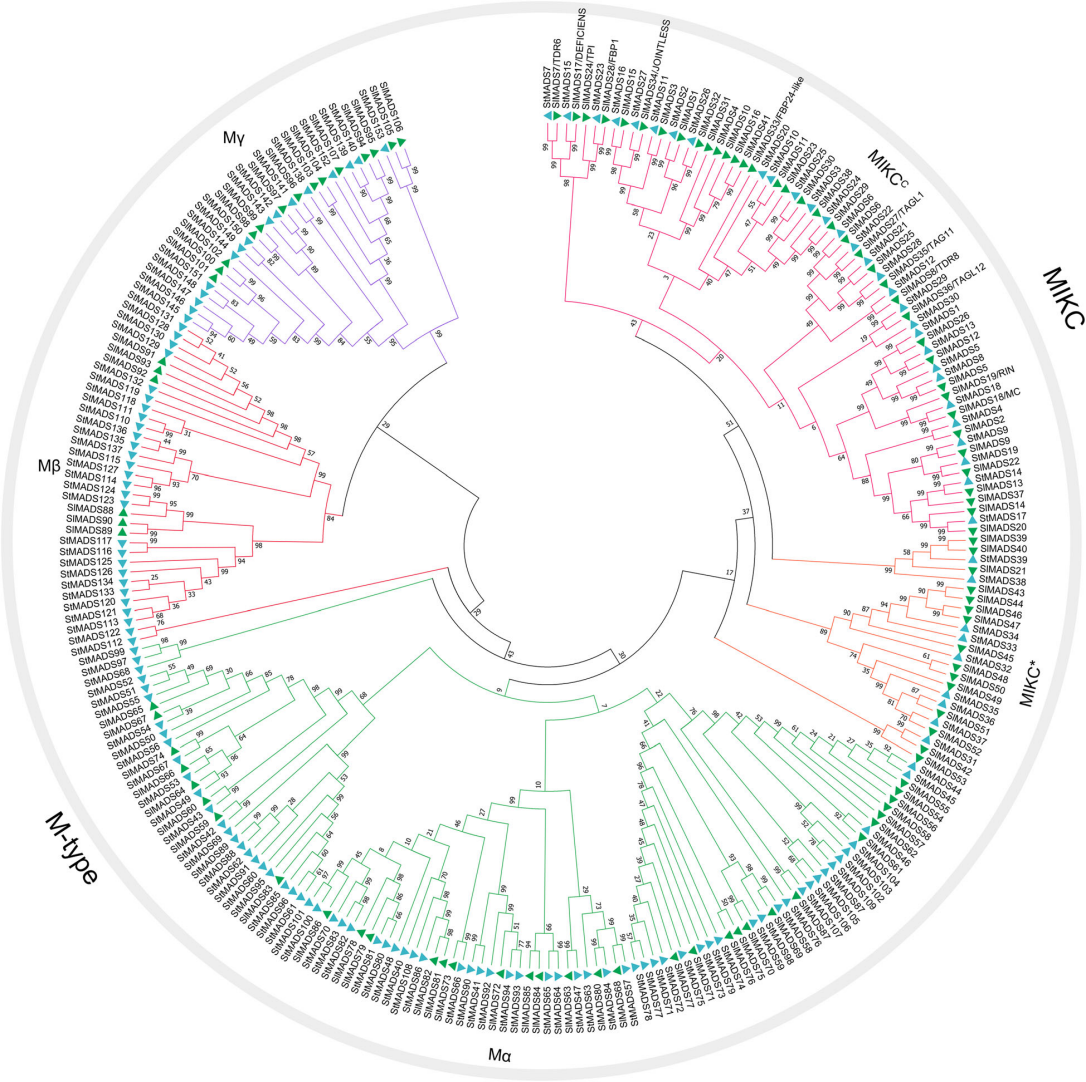
Phylogenetic analyses of MADS-box genes between *Solanum tuberosum* and *Solanum lycopersicum*. The NJ-tree was generated using the method mentioned above

Tomato is the most studied model plant in Solanaceae family. Therefore, a comparison with tomato MADS genes could provide more clues on the function differentiation of potato MADS genes (Fig. 6 and Additional file 2: Table S2). There was a total of 107 MADS-box genes in tomato, including 53 MIKC type and 54 M-type MADS-box genes. Tomato compromised more MIKC genes (53) compared with those in potato (39) even if the total number of potato MADS-box genes (153) is more than those in tomato (107). On the contrary, there were more M-type MADS-box genes in potato compared with those in tomato, especially the number of Mα (70) and Mβ (28) in higher than those in tomato (Mα, 34; Mβ, 6), respectively (Fig. 6 and Table 2). These evidences suggested that the expansion of MADS-box in Solanaceae might be quite different.

To speculate the functions of potato MADS-box genes, we compare the MIKCC MADS-box genes with their closely related homologs. The orthologs of MIKCC potato MADS-box genes was screened by following criteria, which were BLASTP e-Value was less than 10e-10) with more than 80% coverage in length and the ortholog was the best-matching homolog than other candidate in tomato. The orthologs of most potato MIKCC MADS-box genes could be found in tomato except StMADS3, 5, and 20 (Additional file 3: Table S3). The Orthologs in different species have evolved from a common ancestral gene via speciation, which often retain the same functions during evolution.

### Tissue specific expression patterns of MIKC^C^*StMADS* genes

Illumina RNA-Seq transcriptome data of DM and RH was retrieved to explore the expression patterns of *StMADS* genes, including vegetative organs (including root, stem, petiole, and leaf), floral organs (including flower, stamen, sepal, petal, and carpel) and storage organs (including stolon and tuber) [36]. The expression levels of *StMADS* genes were estimated by using FKPM (fragments per kilobase of transcript per million mapped fragment) method. A gene was designated as expressed if its FKPM value in any tissue or organ was greater than 1. According to this standard, 37 of 39 MIKC-type *StMADS* genes were expressed but only 16 of 123 M-type *StMADS* genes were expressed, indicating that MIKC-type *StMADS* genes were actively expressed and most of M-type *StMADS* genes were not expressed even if there were many M-type *StMADS* genes presented on the potato genome.

**Fig. 7:**
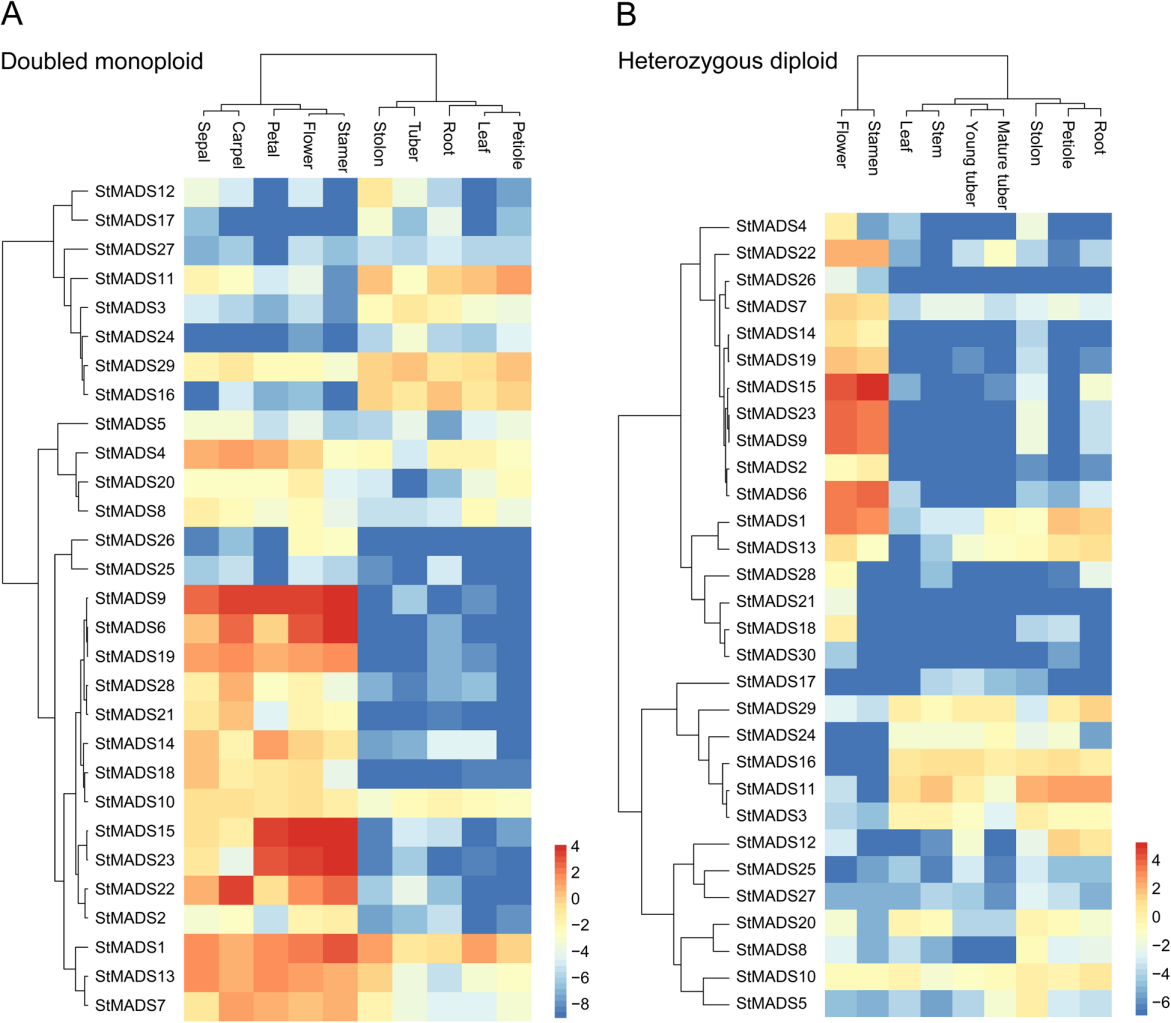
Expression profiles of MADS-box genes in double monoploid and heterozygous. The RNA-seq data is retrieved from PGSC. FPKM values of MADS genes are normalized and the heatmap is drawn with Pretty Heatmap at ImageGP. **a** Expression profiles of the StMADS genes in DM (doubled monoploid S. tuberosum Group PhurejaDM1-3). **b** Expression profiles of the StMADS genes in RH (heterozygous diploid S. tuberosum Group Tuberosum RH89-039-16)

The MIKC^C^ *StMADS* genes were selected for further expression analysis because they were probable downstream targets of tuberigen StSP6A based on previous studies about its homologue−FLOWERING LOCUS T in *Arabidopsis* and rice [34]. Hierachical clustering of MIKC^C^ *StMADS* genes was performed by using the transcriptome data of DM and RH, respectively (Fig. 7). The MIKC^C^ *StMADS* genes in both DM and RH were similarly divided into two major clusters. The first group of MIKC^C^ *StMADS* genes was mainly expressed in floral organs and the other group was expressed in vegetative and storage organs. More specifically for their expressions in DM, 21 genes, five genes, and four genes were with highest expression in floral organs, vegetative organs, and storage organs, respectively (Fig. 7a). And for RH, 16 genes, five genes, and nine genes were with highest expression in floral organs, vegetative organs, and storage organs, respectively (Fig. 7b). It was found that most MIKC^C^ *StMADS* genes were expressed in floral organs, indicating their possible roles in controlling floral organ development. Whereas, there were more MIKC^C^ *StMADS* genes expressed in storage organs of RH compared with DM.

Moreover, the expressions of MIKC^C^ *StMADS* genes in storage organs were further analyzed. It was found that 17 and 26 MIKC^C^ *StMADS* genes were expressed (FKPM > 1) in storage organs of DM and RH, respectively. And nine and eight and 12 MIKC^C^ *StMADS* genes were highly expressed (FKPM > 10) in storage organs of DM and RH, respectively. Taken together, six genes (*StMADS1*, *3*, *11*, *13*, *16*, and *29*) were consistently with high expression levels in storage organs of both DM and RH, which may be involved in tuberization and following tuber development. Based on the phylogenic relationship, it was found that *StMADS1* and *StMADS13* were homologous genes of *AGL8/FUL* and *OsMADS14/15*, *StMADS3* was homologous gene of *SOC1* and *OsMADS56*, *StMADS11* and *StMADS16* were homologous genes of AGL22/SVP, and *StMADS29* was homologous gene of *AGL12*. Among these genes expressed in potato stolon and tubers, *StMADS1*, *StMADS3*, and *StMADS13* were the most likely downstream genes of tuberigen StSP6A because their homologous genes *AGL8/FUL*, *OsMADS14/15*, and *SOC1* were proved to be downstream targets of *Arabidopsis* and rice FLOWERING LOCUS T [53–57].

### QRT-PCR verifications of tissue specific MIKC^C^*StMADS* genes

**Fig. 8:**
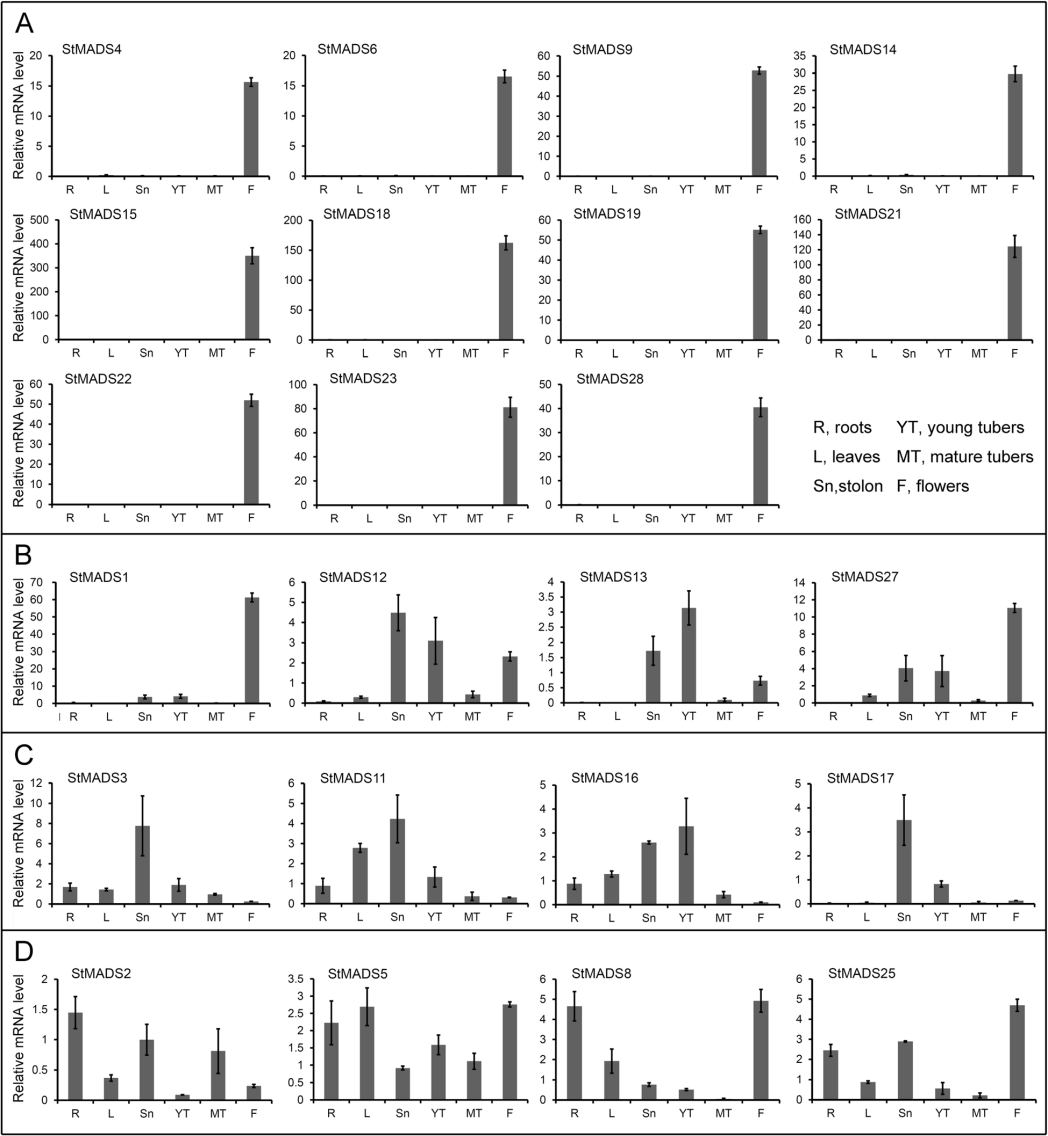
QRT-PCR verifications of representative *StMADS* genes in various potato tissues. **a** Genes are mainly expressed in flowers. **b** Genes preferentially show expression in flowers, stolons, and young tubers. **c** Genes are abundantly expressed in stolons. **d** Genes are expressed in nearly all examined tissues

To validate the results of RNA-seq analysis, real-time PCR analysis was performed for 29 MIKC^C^ *StMADS* genes. Our tests showed that the real-time PCR experiments of 25 MIKC^C^ *StMADS* genes (except *StMADS7*, *10*, *20*, and *26*) were successfully conducted in tissues including roots, leaves, stolons, young tubers, mature tubers, and flowers. The results of real-time PCR showed that the expression patterns of most were in general agreement with the data of RNA-seq analysis. For example, eleven *StMADS* genes (*StMADS4*, *6*, *9*, *14*, *15*, *18*, *19*, *21*, *22*, *23*, and *28*) were overwhelmingly expressed in flowers compared with any other tissues (Fig. 8a), which were perfectly consistent with their expression patterns in flowers of both DM and RH (Fig. 7). The *StMADS* genes specifically expressed in potato flowers were most likely to control floral organ formation like their homologues in ABCDE model of other species [58, 59].

*StMADS1*, *12*, *13*, and *27* were not only expressed in flowers but also expressed in stolons and young tubers (Fig. 8b), indicating that they might control the formations of both flower organs and tuberization. *StMADS3*, *11*, *16*, and *17* were highly expressed in stolons and/or young tubers but their expressions in flower were relatively low (Fig. 8c). Besides, we found six *StMADS* genes were expressed in almost all examined tissues without obvious tissue specific patterns (Fig. 8d, the expressions of *StMADS24* and *StNADS29* were not showed.

### Screening for downstream targets of tuberigen StSP6A

StSP6A, a FLOWERING LOCUS T homologue in potato, have been reported to be a mobile signal in controlling not only flowering but also tuberization, while its homologue StSP3D is mainly involved in floral transition [34]. According to previous studies about flowering, MADS-box genes encoding proteins involved in flowering identity determination are major targets of FLOWERING LOCUS T and it was speculated that *StMADS* genes were downstream targets of tuberigen StSP6A. Therefore, the whole-genome microarray data from stolon tissue of *StSP6A*-overexpression (*StSP6A*-OX) and *StSP6A*-RNAi plants was used to screen for downstream *StMADS* genes. Firstly, the DNA probe sequences on POCI (Potato Oligo Chip Initiative) [34] microarrays were used as queries to perform BLASTN searches against the transcript sequences of *StMADS* genes. It was found that 22 probes corresponded to 17 *StMADS* genes (16 genes were belonged to MIKC^C^ type) were presented on the POCI microarray chip and four genes had two or three probes. Hierachical clustering of the expressions of *StMADS* genes in StSP6A-OX and StSP6A-RNAi plants showed that StMADS1 and StMADS13 were the most possible downstream targets of StSP6A because the expressions of *StMADS1* and *StMADS13* were activated in both biological replicates of *StSP6A*-OX plants and was repressed in both biological replicates of *StSP6A*-RNAi plants. The expressions of *StMADS3* and *StMADS11* were only activated in one biological replicate of *StSP6A*-OX plants and not repressed in *StSP6A*-RNAi plants. The expressions of other *StMADS* genes were not obviously changed in either *StSP6A*-OX or *StSP6A*-RNAi plants.

**Fig. 9:**
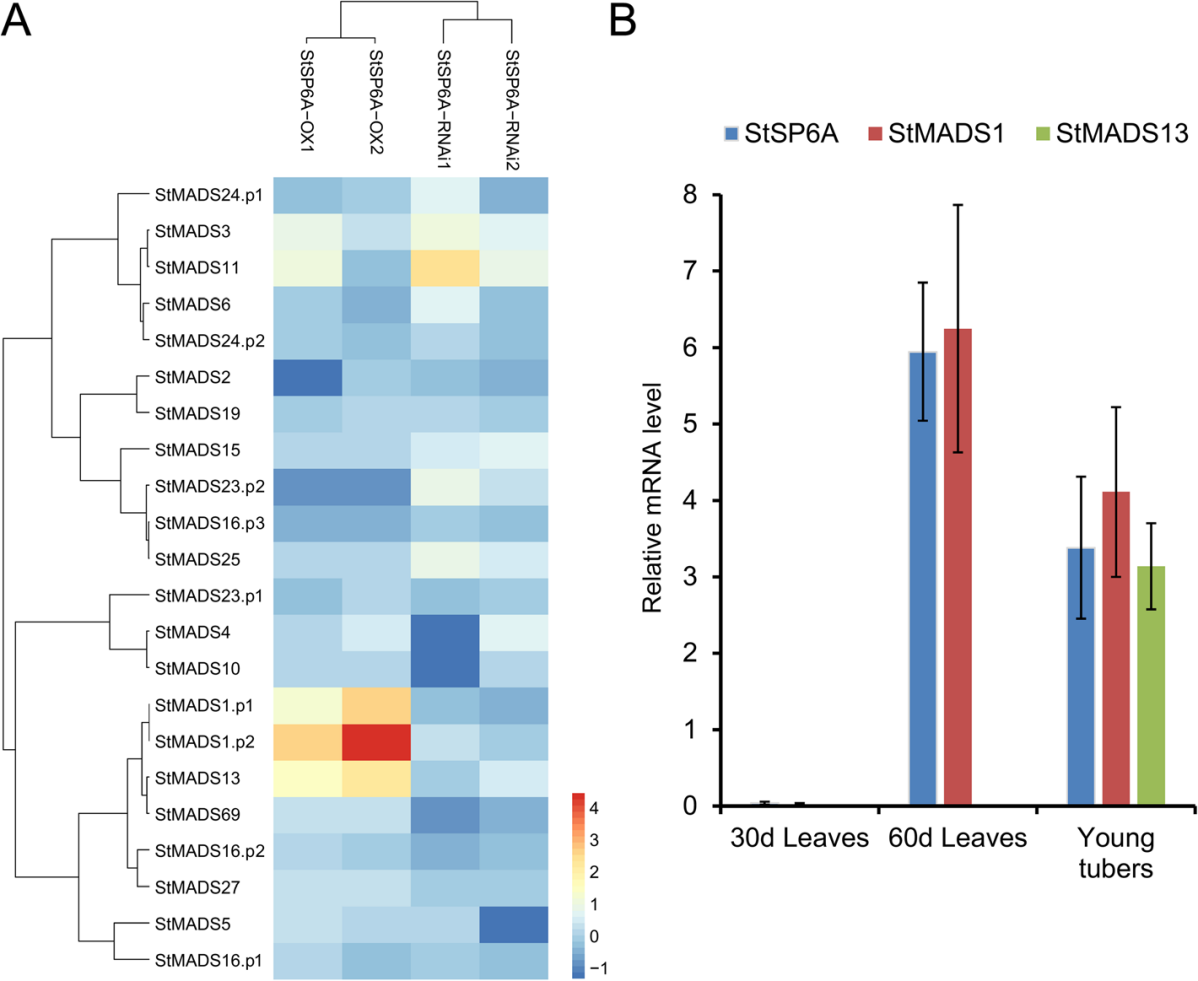
Expression relationships between MADS-box genes and *StSP6A*. **a** Heatmap of the expression of 17 MADS-box genes in *StSP6A*-OX and *StSP6A*-RNAi plants. **b** Expressions of *StSP6A*, *StMADS1*, and *StMADS13* in leaves of 30 days after sprouting, leaves of 60 days after sprouting, and young tubers, respectively

Moreover, to verify the whether the expressions of *StAMDS1* and *StMADS13* were associated with the expression of *StSP6A*, we investigate the expressions of *StSP6A*, *StMADS1*, and *StMADS13* in leaves of 30 days after sprouting, leaves of 60 days after sprouting, and young tubers, respectively. It was found that these three genes were not expressed in potato leaves at juvenile stage (30 days after sprouting), whereas *StMADS1* and *StSP6A* were highly expressed in potato leaves at early flowering stage (60 days after sprouting) and young tubers (Fig. 9b). The expression of *StMADS13* was not detected in 60d leaves but was observed in young tubers (Fig. 9b). These results indicated that *StMADS1* expressions were associated with *StSP6A* in both 60d leaves and young tubers, whereas *StMADS13* was only associated with *StSP6A* in young tubers. Though both *StMADS1* and *StMADS13* were putative downstream genes of *StSP6A*, their regulatory mechanism might be different depending on tissue types.

## Discussion

Potato is one of the major food crops, which feeds millions of people all over the world [60]. As an important tuber crop, the improvement of yield is a key issue to potato breeder in china, owing to its low production far fewer than the global average. Thus, the investigation of molecular mechanism of tuberization and tuber development remains unclear. It will be helpful to identify candidate genes related to tuberization and tuber development, which are the key resources to promote the improvement of yield for both genetic modified crop and traditional breeding. Previous study had shown that FT protein StSP6A functioned as a mobile signal in controlling tuberization under short-day condition [34], and its paralogue StSP3D was involved in day-neutral flowering control. FT was a classical upstream regulator of MADS-box genes in the conservative ABC model in flower organ identity [59]. Based on clues that we mentioned above, it was reasonable to believe that some potato MADS-box genes were related to tuberization and tuber development.

In this study, a total of 153 members of MADS-box gene family were characterized in potato (Table 1). To confirm the gain and loss of MADS-box genes in potato, a phylogenetic tree was produced using ammo acid sequence of 153 potato MADS-box genes and representative MADS genes of *Arabidopsis* and rice (Fig. 1). It was found that most of MADS-box genes had their orthologs in potato, except for TT16-like, AGL17-like, and FLC-like subgroups. Besides, it was found that members of M-type, specifically Mα, were much more than any other species ever studied (Table 2), implying that potato MADS-box genes might have a different evolution pattern [39–52]. Apparently, the large potato MADS-box family might be due to its larger genome, which was produced by differently genomic duplication events in different species during the course of plant evolution [61, 62]. To explore what behind the extremely different composition of MADS-box family in potato, the effects of gene duplication events on expansion of MADS-box genes were investigated (Fig. 2). It was suggested that segmental duplications mainly contributed to expansion of MIKC subfamily, whereas the boom of M-type was mostly derived from tandem duplications (Figs. 2 and 3). It was believed that this phenomenon was due to the M-type genes mainly derived from the site-specific duplications within the same chromosome, while MIKC mainly came from the whole genome duplication events [61, 63–65]. Birth-and-death rates of MADS-box genes after gene duplications in different species were different which resulted in variable number of MADS-box genes in the same subfamily of different species [61, 62]. These evidences could be one explanation for the abnormality of number of Mα in potato.

Besides gene duplication events, gene mutation and loss of certain domain, might also play important role in generating a part of Mα in potato. It had been reported that MIKC^*^ was the intermediate form between MIKC^C^ and M-type in the course of plant evolution [58, 59, 62]. Based on intron-exon structures of potato MADS-box genes, it was found that the intron number of MIKC^C^ was the most and MIKC* had less intron than MIKC^C^, whereas most of M-type MADS-box genes was intronless. It could be speculated that the exon-intron loss mutations mainly happened in the K-box domain of MIKC^C^ in the process of plant evolution, thus a new group, MIKC^*^, was born. MIKC^*^ further lost several exons and introns corresponding to K-box domain and then produced M-type, which was also found in previous studies [49]. These evidences could be the other explanation for the abnormality of number of Mα in potato.

To compare the composition of MADS-box genes of potato and tomato, phylogenetic analysis was performed. Since these two species are genetically close species, it was surprising that tomato contain much more MIKC genes, while potato included more M-type genes. This suggested that these species might undergo a different evolution history of MADS-box. The candidate genes *StMADS1* and *StMADS13* that may be related to tuberization, the functions of their orthologous genes in tomato, *SlMADS26* and *SlMADS12*, were still undiscovered. Though potato and tomato are both Solanaceae plants, the stolons and tubers are only found in potato. Thus, the functions of *StMADS1* and *StMADS13* need to be investigated in potato. Nevertheless, there was a homolog of *StMADS1* and *StMADS13*, RIN found in tomato, which is proved to play important roles in induction of tomato ripening [66, 67]. Besides, compared with potato, most genes that had been studied in tomato have few homologs, indicating that a different history of gain and loss functions of MADS-box genes [68–70].To investigate the possible roles of *StMADS* genes in tuberization, we used RNA-seq data of DH and RM available in PGSC. Most of the *StMADS* genes showed tissue-specific expression patterns. Among these genes, *StMADS1*, *3*, *11*, *13*, *16*, and *29* were highly expressed in storage organs of both DM and RH (Fig. 7). Consistent with RNA-seq data, the results of QRT-PCR showed that *StMADS1*, *3*, *11* and *16* were overwhelmingly expressed in stolons and/or young tubers (Fig. 8), indicating that these genes were probably involved in tuberization and/or tuber development. Previous studies had shown us a fine regulation map of MADS-box genes and its significant roles in flower organ differentiation in several model plants. In *Arabidopsis*, the expression of *FLOWERING LOCUS T* (*FT*), a core flower development regulator, was suppressed by the *FLOWERING LOCUS C* (*FLC*), a typical MADS-box genes, bound in its CArG site between first intron and promoter [71–73]. Interestingly, the expressions of MADS-box genes including *APETALA1* (*AP1*) and *SUPPRESSOR OF OVEREXPRESSION OF CO 1*(*SOC1*) were related to flowering promotion that was controlled by two interacted flowering-related proteins FT and FD (*FLOWERING LOCUS D*) [74]. In monocot plants, orthologs and paralogs of FT and MADS-box presented nearly the same transcriptional regulation, for instance, a pair of FT genes *Heading-date 3a* (*Hd3a*) and *RICE FLOWERING LOCUS T* (*RFT1*) upregulated the expression of *OsMADS15*, which is crucial for floral initiation [75–78]. Given the highly conservative model of FT and MADS-box genes, it was reasonable to believe that this model would probably work in potato. Recent study showed that there was a functional diversification of FT proteins in potato. StSP3D was mainly involved in floral transition, and StSP6A was involved in tuberization transition. [34]. Therefore, these *StMADS* genes (*StMADS1*, *3*, *11*–*13*, 17, and *27*) mainly expressed in stolons and/or young tubers were possible downstream targets of StSP6A. Interestingly, it was found *StMADS1* and *13* were strongly correlated with the expression of *StSP6A* in leaves and/or young tubers. More evidences were obtained through analyzing the microarray data from stolon tissue of StSP6A-overexpression (*StSP6A*-OX) and *StSP6A*-RNAi plants, the expression of *StMADS1* and *13* were upregulated in *StSP6A*-OX plants and downregulated in *StSP6A*-RNAi plants. Given the evidence that discussed above, *StSP6A* and several MADS-box genes are probably share the same regulation map with their homologs in the model plant. However, the truth of how *StSP6A* regulate *StMADS1* and *13*, in a directly interaction way or in the promotor region, remain unclear. As a master of transcription, tracing the target of MADS-box gene would also be a valuable subject in the future study.

## Conclusion

This study present genomic annotation and expression profiling of potato MADS-box genes. Comprehensive analyses about the evolution and functional differentiation of potato MADS-box were also performed, which would provide solid basis for further functional studies about this gene family. Potato MADS-box genes were putative downstream targets of the potato FT homolog tuberigen StSP6A, which is a mobile signal in controlling tuberization. Furthermore, *StMADS1* and *StMADS13* were believed to be candidate genes in the downstream of StSP6A. Thus, the utilization of these MADS-box genes for both genetically modified crop and traditional breeding practice in genetic improvement would be possible.

## Additional files

Additional file 1: **Table S1.** QRT-PCR primers corresponding to potato MADS-box genes. (DOCX 18 kb)

Additional file 2: **Table S2.** MADS-box genes in tomato. (DOCX 25 kb)

Additional file 3: **Table S3.** Orthologs of potato MIKC^C^ MADS-box genes in tomato. (DOCX 18 kb)

## Abbreviations

AA: Amino acid
AP1: APETALA1
BLASTP: Basic local alignment search tool-protein
FLC: FLOWERING LOCUS C
FT: FLOWERING LOCUS T
Hd3a: Heading-date 3a
ORF: Open reading frame
PGDD: Plant genome duplication database
PGSC: Potato genome sequencing consortium
QRT-PCR: Quantitative real-time polymerase chain reaction
RFT1: RICE FLOWERING LOCUS T
SOC1: SUPPRESSOR OF OVEREXPRESSION OF CO 1
