# Supplementary material for: Genome-wide survey of potato MADS-box genes reveals that StMADS1 and StMADS13 are putative downstream targets of tuberigen StSP6A
Source: BMC Genomics. 2018 Oct 3;19:726. doi: 10.1186/s12864-018-5113-z (PMC6171223; doi:10.1186/s12864-018-5113-z)
Supplement: Supplementary file 1 — Table S1. QRT-PCR primers corresponding to potato MADS-box genes. (DOCX 18 kb) [file 12864_2018_5113_MOESM1_ESM.docx]

| **Gene** | **Forward Primer (5'-3')** | **Reverse Primer (5'-3')** |
| --- | --- | --- |
| StMADS1 | AAATGGGAAGAGGAAGAGT | GAAGGTACGTTTAGACAAT |
| StMADS2.p1 | TGCCGTTTCCAGAGTTTCG | CTCTAGGCACCATAATCAG |
| StMADS2.p2 | CCCCTCTTCCTAATTTCTT | AACAAGTTACAAAGGAGGG |
| StMADS3 | TTTAGCTCCAATAGTCCTT | AACCCACTCATACATCTCC |
| StMADS4 | CCACCTTACCTTGTTTCTC | GAATTAGAAATTAAAGACCC |
| StMADS5.p1 | ACTCGTTCTAAATCCATCT | ATTACTACAACAAAGCCAT |
| StMADS5.p2 | TGTTAGATTAGAACTCGTT | GCTCATCATCTGCTTCATA |
| StMADS6.p1 | AATACCTATCCCTTAGTCC | TCATAGTTTACTGCCTGAA |
| StMADS6.p2 | CTCTTTCCTTTCCTTACAA | TCAGTTCCAATAGTCTTCA |
| StMADS7.p1 | TCTACTAACCCACCCATCT | TTCAAGGCATCACCCATAT |
| StMADS7.p2 | TGTTTCATCACCTCCATTA | GCTTAGCTCCATTGATGAC |
| StMADS8.p1 | TCACATGGTAGGGAACAAC | TAAGGAGGGATGGATAAAA |
| StMADS8.p2 | TTATTGTGCCCTTTCACTC | ATGCGCTGCTTAAATCTGT |
| StMADS9.p1 | TTTGGTTGTTTCAGTAGGA | AGATAAGGGAGCGTAGGAC |
| StMADS9.p2 | ATCCCTAAAGAAGATAAGA | CGATAACATTTTGCTGAAC |
| StMADS10 | ATGGTGAGAGGAAAAGTAGA | TCAAAGGCGGAAGGTCTGAG |
| StMADS11.p1 | TCTTCTCCATTTCTTGCAA | ATTAGACCGTTGACCCCTC |
| StMADS11.p2 | TTCAGGGTGAAGAAAGAAA | CTAATTCAACATTCGATAAG |
| StMADS12 | TCATTTGAAGAATGGGGAGA | GGCTCTTTAATTACACGAC |
| StMADS13 | TGCCAAACAAAGAAATACT | TATTGTCCGCTTGGTTTAT |
| StMADS14 | GCTTGTAACCAATAATCCC | CGATAATCCTTGAACTAAAT |
| StMADS15.p1 | CCTTTTAGGGTTTGAGTAT | CATTGCATGTGATTATTTAG |
| StMADS15.p2 | ACTCTTCTCCTCCTCCAAT | CATGACACTTCAATCCAAA |
| StMADS16.p1 | AGCATCCAACCACTCCCTC | CTGCATTATGCCCTTTGTC |
| StMADS16.p2 | TAACACGGCTTCTCAACAG | ATTATGCCCTTTGTCAAGT |
| StMADS17 | ATGGGAAGAGGGAAAGTAG | CATACTCCAAATTCAAAGC |
| StMADS18 | TGTAGAAAAGAAGTAAAGT | CATCTGTCCTGATAGTGAG |
| StMADS19 | AAGGGGTTTTGTGCTTTTG | TAATGGGATTGTCTTATGG |
| StMADS20 | ATGATTAACGTCTATTTCAA | TCAGACCACGGCCCGTGCT |
| StMADS21 | TTTCAATTCAAGCCAATCA | TTCTTACAAACCCCAAAAC |
| StMADS22 | ATGGTTTTTCCTAATAATCA | TTAAGAAGGAAGAGAAGCAT |
| StMADS23 | ATGGGGAGAGGAAAAATAGA | CATTATCCACCGAGTTTAG |
| StMADS24 | CCTTTGAACCAATTTTGTT | TAAAACTTGAAACACCAGC |
| StMADS25 | ATGGTGAGAGGGAAAACTGA | AAGGCCAAAGAGGAAACGA |
| StMADS26 | AATTAAACCAAGACTCTTT | ATGTGATGGTCTTACAACT |
| StMADS27.p1 | CCCTCTTCCTTCTTAACTC | CCTTCATCCGATAACATTC |
| StMADS27.p2 | GTTTATTCTTTGTTCCCTC | GGAGACAAATTCATGGGAT |
| StMADS28 | ATGGGTCGAGGAAAGATAGA | TTCCTCCACAGTAGAAATG |
| StMADS29 | ATGGCTCGTGGTAAGGTTCA | CTATGTACTGACGCCATTTG |
| StMADS30 | ATGGGAAGAGGTAAGGTAGA | TTATTGCACCAAATAGGGCA |

**Table S1.** qRT-PCR primers corresponding to potato MADS-box genes
