# Supplementary material for: Genome-wide survey of potato MADS-box genes reveals that StMADS1 and StMADS13 are putative downstream targets of tuberigen StSP6A
Source: BMC Genomics. 2018 Oct 3;19:726. doi: 10.1186/s12864-018-5113-z (PMC6171223; doi:10.1186/s12864-018-5113-z)
Supplement: Supplementary file 2 — Table S2. MADS-box genes in tomato. (DOCX 25 kb) [file 12864_2018_5113_MOESM2_ESM.docx]

**Table S2.** MADS-box genes in tomato

| **Gene Name** | **Gene ID** | **Chromosome** | **Location** | **Subfamily** |
| --- | --- | --- | --- | --- |
| SlMADS1 | XP_010324817 | Chr01 | 82704879-82711143 | MIKC^C^ |
| SlMADS2 | NP_001348459 | Chr01 | 85410512-85415751 | MIKC^C^ |
| SlMADS3 | XP_004230624 | Chr01 | 93734559-93742594 | MIKC^C^ |
| SlMADS4 | XP_019067399 | Chr01 | 94008522-94020923 | MIKC^C^ |
| SlMADS5 | XP_010316206 | Chr02 | 37449600-37454150 | MIKC^C^ |
| SlMADS6 | XP_010316397 | Chr02 | 48350863-48354641 | MIKC^C^ |
| SlMADS7/TDR6 | NP_001234105 | Chr03 | 62900860-62904420 | MIKC^C^ |
| SlMADS8/TDR8 | NP_001295225 | Chr02 | 41661660-41667474 | MIKC^C^ |
| SlMADS9 | XP_010315869 | Chr02 | 51712279-51716842 | MIKC^C^ |
| SlMADS10 | XP_025885553 | Chr02 | 53447063-53454713 | MIKC^C^ |
| SlMADS11 | NP_001276829 | Chr03 | 1331833-1343622 | MIKC^C^ |
| SlMADS12 | NP_001294867 | Chr03 | 66182952-66189463 | MIKC^C^ |
| SlMADS13 | NP_001234380 | Chr03 | 66198833-66205137 | MIKC^C^ |
| SlMADS14 | XP_004237061 | Chr04 | 228275-233497 | MIKC^C^ |
| SlMADS15 | XP_004237993 | Chr04 | 61289076-61295146 | MIKC^C^ |
| SlMADS16 | XP_025886615 | Chr04 | 61665583-61689585 | MIKC^C^ |
| SlMADS17/DEFICIENS | NP_001234077 | Chr04 | 65145926-65149587 | MIKC^C^ |
| SlMADS18/MC | NP_001234665 | Chr05 | 5217005-5222574 | MIKC^C^ |
| SlMADS19/RIN | NP_001233976 | Chr05 | 5217005-5231124 | MIKC^C^ |
| SlMADS20 | NP_001234670 | Chr05 | 5225417-5230937 | MIKC^C^ |
| SlMADS21 | XP_010321014 | Chr05 | 11818209-11841445 | MIKC^C^ |
| SlMADS22 | NP_001234384 | Chr05 | 11897272-11903607 | MIKC^C^ |
| SlMADS23 | XP_004240747 | Chr06 | 24636174-24672466 | MIKC^C^ |
| SlMADS24/TPI | NP_001234075 | Chr06 | 38012808-38015675 | MIKC^C^ |
| SlMADS25 | XP_025887358 | Chr06 | 40536100-40543185 | MIKC^C^ |
| SlMADS26 | XP_019069686 | Chr06 | 43291464-43299544 | MIKC^C^ |
| SlMADS27/TAGL1 | NP_001300859 | Chr07 | 63971953-63981792 | MIKC^C^ |
| SlMADS28/FBP1 | XP_004245202 | Chr08 | 56323333-56328094 | MIKC^C^ |
| SlMADS29 | XP_004245665 | Chr08 | 63541431-63552923 | MIKC^C^ |
| SlMADS30 | XP_025883671 | Chr10 | 5566688-5572700 | MIKC^C^ |
| SlMADS31 | XP_004248967 | Chr10 | 29071774-29081560 | MIKC^C^ |
| SlMADS32 | XP_004249753 | Chr10 | 61570359-61573439 | MIKC^C^ |
| SlMADS33/FBP24-like | XP_019066630 | Chr11 | 107104-109736 | MIKC^C^ |
| SlMADS34/JOINTLESS | NP_001306770 | Chr11 | 3640914-3646080 | MIKC^C^ |
| SlMADS35/TAG11 | NP_001234194 | Chr11 | 20042780-20051324 | MIKC^C^ |
| SlMADS36/TAGL12 | NP_001233764 | Chr11 | 25030835-25050985 | MIKC^C^ |
| SlMADS37 | NP_001275579 | Chr12 | 51384218-51396364 | MIKC^C^ |
| SlMADS38 | XP_004252597 | Chr12 | 63354911-63364960 | MIKC^C^ |
| SlMADS39 | XP_004252711 | Chr12 | 64289726-64302926 | MIKC^C^ |
| SlMADS40 | XP_004252712 | Chr12 | 64306063-64318145 | MIKC^C^ |
| SlMADS41 | XP_004252725 | Chr12 | 64547844-64559507 | MIKC^C^ |
| SlMADS42 | XP_025885911 | Chr03 | 66977099-66980837 | MIKC* |
| SlMADS43 | XP_019069043 | Chr04 | 52650737-52655899 | MIKC* |
| SlMADS44 | XP_025886282 | Chr04 | 52663528-52669064 | MIKC* |
| SlMADS45 | XP_019068982 | Chr04 | 52748668-52751451 | MIKC* |
| SlMADS46 | XP_025886283 | Chr04 | 52880876-52886547 | MIKC* |
| SlMADS47 | XP_025886284 | Chr04 | 52955386-52961060 | MIKC* |
| SlMADS48 | XP_010320151 | Chr04 | 59770985-59784437 | MIKC* |
| SlMADS49 | XP_010320313 | Chr04 | 63144066-63147659 | MIKC* |
| SlMADS50 | XP_004239996 | Chr05 | 63081398-63087508 | MIKC* |
| SlMADS51 | XP_004243447 | Chr07 | 61284407-61290723 | MIKC* |
| SlMADS52 | XP_019070464 | Chr07 | 61292192-61305889 | MIKC* |
| SlMADS53 | XP_025883950 | Chr11 | 31267373-31277409 | MIKC* |
| SlMADS54 | XP_004229071 | Chr01 | 69957477-69958070 | Mα |
| SlMADS55 | XP_004229070 | Chr01 | 69998147-69998740 | Mα |
| SlMADS56 | XP_010319771 | Chr01 | 70032677-70035118 | Mα |
| SlMADS57 | XP_004229069 | Chr01 | 70082253-70082846 | Mα |
| SlMADS58 | XP_004229068 | Chr01 | 70100941-70101534 | Mα |
| SlMADS59 | XP_004229320 | Chr01 | 74303433-74304362 | Mα |
| SlMADS60 | XP_025886320 | Chr01 | 74707434-74708506 | Mα |
| SlMADS61 | XP_004229572 | Chr01 | 79895656-79896249 | Mα |
| SlMADS62 | XP_004229573 | Chr01 | 79909763-79910356 | Mα |
| SlMADS63 | XP_004229956 | Chr01 | 86275288-86276251 | Mα |
| SlMADS64 | XP_010315464 | Chr01 | 88527439-88527921 | Mα |
| SlMADS65 | XP_004231248 | Chr01 | 88533826-88534284 | Mα |
| SlMADS66 | XP_004231249 | Chr01 | 88537835-88538287 | Mα |
| SlMADS67 | XP_004231250 | Chr01 | 88542519-88543525 | Mα |
| SlMADS68 | XP_004231377 | Chr01 | 94362371-94363485 | Mα |
| SlMADS69 | XP_004231378 | Chr01 | 94363606-94364625 | Mα |
| SlMADS70 | XP_004231379 | Chr01 | 94366507-94367622 | Mα |
| SlMADS71 | XP_004231380 | Chr01 | 94370609-94371397 | Mα |
| SlMADS72 | XP_025886043 | Chr13 | 1565115-1565612 | Mα |
| SlMADS73 | XP_004234621 | Chr13 | 5992296-5992850 | Mα |
| SlMADS74 | XP_004234880 | Chr13 | 34538849-34539286 | Mα |
| SlMADS75 | XP_004237288 | Chr04 | 25534673-25535065 | Mα |
| SlMADS76 | XP_004237284 | Chr04 | 26206213-26206620 | Mα |
| SlMADS77 | XP_004237686 | Chr04 | 38145440-38146012 | Mα |
| SlMADS78 | XP_004237586 | Chr04 | 54440742-54441400 | Mα |
| SlMADS79 | XP_004237582 | Chr04 | 54749924-54750582 | Mα |
| SlMADS80 | XP_004237763 | Chr04 | 56040518-56042255 | Mα |
| SlMADS81 | XP_004241160 | Chr06 | 37554099-37554799 | Mα |
| SlMADS82 | XP_010323002 | Chr06 | 37793848-37794324 | Mα |
| SlMADS83 | XP_004247303 | Chr09 | 61017659-61018180 | Mα |
| SlMADS84 | XP_004248619 | Chr10 | 6634320-6634874 | Mα |
| SlMADS85 | XP_019071580 | Chr10 | 6669234-6670031 | Mα |
| SlMADS86 | XP_004248898 | Chr10 | 51143131-51143616 | Mα |
| SlMADS87 | XP_019066402 | Chr11 | 54691310-54697680 | Mα |
| SlMADS88 | XP_019066449 | Chr11 | 10863773-10865014 | Mβ |
| SlMADS89 | XP_010312953 | Chr11 | 11776332-11777360 | Mβ |
| SlMADS90 | XP_004250617 | Chr11 | 11829495-11831299 | Mβ |
| SlMADS91 | XP_010313957 | Chr12 | 6005008-6006363 | Mβ |
| SlMADS92 | XP_004252136 | Chr12 | 6233114-6233859 | Mβ |
| SlMADS93 | XP_019066913 | Chr12 | 6258058-6259040 | Mβ |
| SlMADS94 | XP_010319860 | Chr01 | 68995447-68996562 | Mγ |
| SlMADS95 | XP_010319833 | Chr01 | 69103491-69104603 | Mγ |
| SlMADS96 | XP_004234709 | Chr03 | 7237266-7238168 | Mγ |
| SlMADS97 | XP_010319640 | Chr04 | 18901967-18903136 | Mγ |
| SlMADS98 | XP_004240285 | Chr05 | 59531163-59531915 | Mγ |
| SlMADS99 | XP_010321641 | Chr05 | 59614123-59614875 | Mγ |
| SlMADS100 | XP_010321633 | Chr05 | 60130876-60131835 | Mγ |
| SlMADS101 | XP_010321632 | Chr05 | 60144157-60148673 | Mγ |
| SlMADS102 | XP_010321635 | Chr06 | 60087780-60088739 | Mγ |
| SlMADS103 | XP_004243902 | Chr07 | 56731771-56732418 | Mγ |
| SlMADS104 | XP_004243899 | Chr07 | 56782803-56783450 | Mγ |
| SlMADS105 | XP_019066947 | Chr12 | 59459051-59459992 | Mγ |
| SlMADS106 | XP_010314444 | Chr12 | 59463471-59464082 | Mγ |
| SlMADS107 | XP_010314958 | Un | 75-1410 | Mγ |
