# Supplementary material for: Genome-wide survey of potato MADS-box genes reveals that StMADS1 and StMADS13 are putative downstream targets of tuberigen StSP6A
Source: BMC Genomics. 2018 Oct 3;19:726. doi: 10.1186/s12864-018-5113-z (PMC6171223; doi:10.1186/s12864-018-5113-z)
Supplement: Supplementary file 3 — Table S3. Orthologs of potato MIKCC MADS-box genes in tomato. (DOCX 18 kb) [file 12864_2018_5113_MOESM3_ESM.docx]

| **Potato MADS** | | **Orthologs in tomato** |  |
| --- | --- | --- | --- |
| StMADS1 | SlMADS26 | | |
| StMADS2 | SlMADS1 | | |
| StMADS3 | Not available | | |
| StMADS4 | SlMADS2 | | |
| StMADS5 | Not available | | |
| StMADS6 | SlMADS6 | | |
| StMADS7 | SlMADS7/TDR6 | | |
| StMADS8 | SlMADS5 | | |
| StMADS9 | SlMADS9 | | |
| StMADS10 | SlMADS11 | | |
| StMADS11 | SlMADS3 | | |
| StMADS12 | SlMADS8/TDR8 | | |
| StMADS13 | SlMADS12 | | |
| StMADS14 | SlMADS13 | | |
| StMADS15 | SlMADS17/DEFICIENS | | |
| StMADS16 | SlMADS15 | | |
| StMADS17 | SlMADS20 | | |
| StMADS18 | SlMADS18/MC | | |
| StMADS19 | SlMADS22 | | |
| StMADS20 | Not available | | |
| StMADS21 | SlMADS25 | | |
| StMADS22 | SlMADS27/TAGL1 | | |
| StMADS23 | SlMADS28/FBP1 | | |
| StMADS24 | SlMADS29 | | |
| StMADS25 | SlMADS30 | | |
| StMADS26 | SlMADS32 | | |
| StMADS27 | SlMADS34/JOINTLESS | | |
| StMADS28 | SlMADS35/TAG11 | | |
| StMADS29 | SlMADS36/TAGL12 | | |

**Table S3.** Orthologs of potato MIKC^C^ MADS-box genes in tomato
